# Materials science in the era of large language models: a perspective†

**DOI:** 10.1039/d4dd00074a

**Published:** 2024-06-05

**Authors:** Ge Lei, Ronan Docherty, Samuel J. Cooper

**Affiliations:** a Dyson School of Design Engineering, Imperial College London London SW7 2DB UK samuel.cooper@imperial.ac.uk; b Department of Materials, Imperial College London London SW7 2DB UK

## Abstract

Large Language Models (LLMs) have garnered considerable interest due to their impressive natural language capabilities, which in conjunction with various emergent properties make them versatile tools in workflows ranging from complex code generation to heuristic finding for combinatorial problems. In this paper we offer a perspective on their applicability to materials science research, arguing their ability to handle ambiguous requirements across a range of tasks and disciplines means they could be a powerful tool to aid researchers. We qualitatively examine basic LLM theory, connecting it to relevant properties and techniques in the literature before providing two case studies that demonstrate their use in task automation and knowledge extraction at-scale. At their current stage of development, we argue LLMs should be viewed less as oracles of novel insight, and more as tireless workers that can accelerate and unify exploration across domains. It is our hope that this paper can familiarise materials science researchers with the concepts needed to leverage these tools in their own research.

## Abstract 2

This perspective paper explores the potential of Large Language Models (LLMs) in materials science, highlighting their abilities to handle ambiguous tasks, automate processes, and extract knowledge at scale across various disciplines.

## Graphical abstract

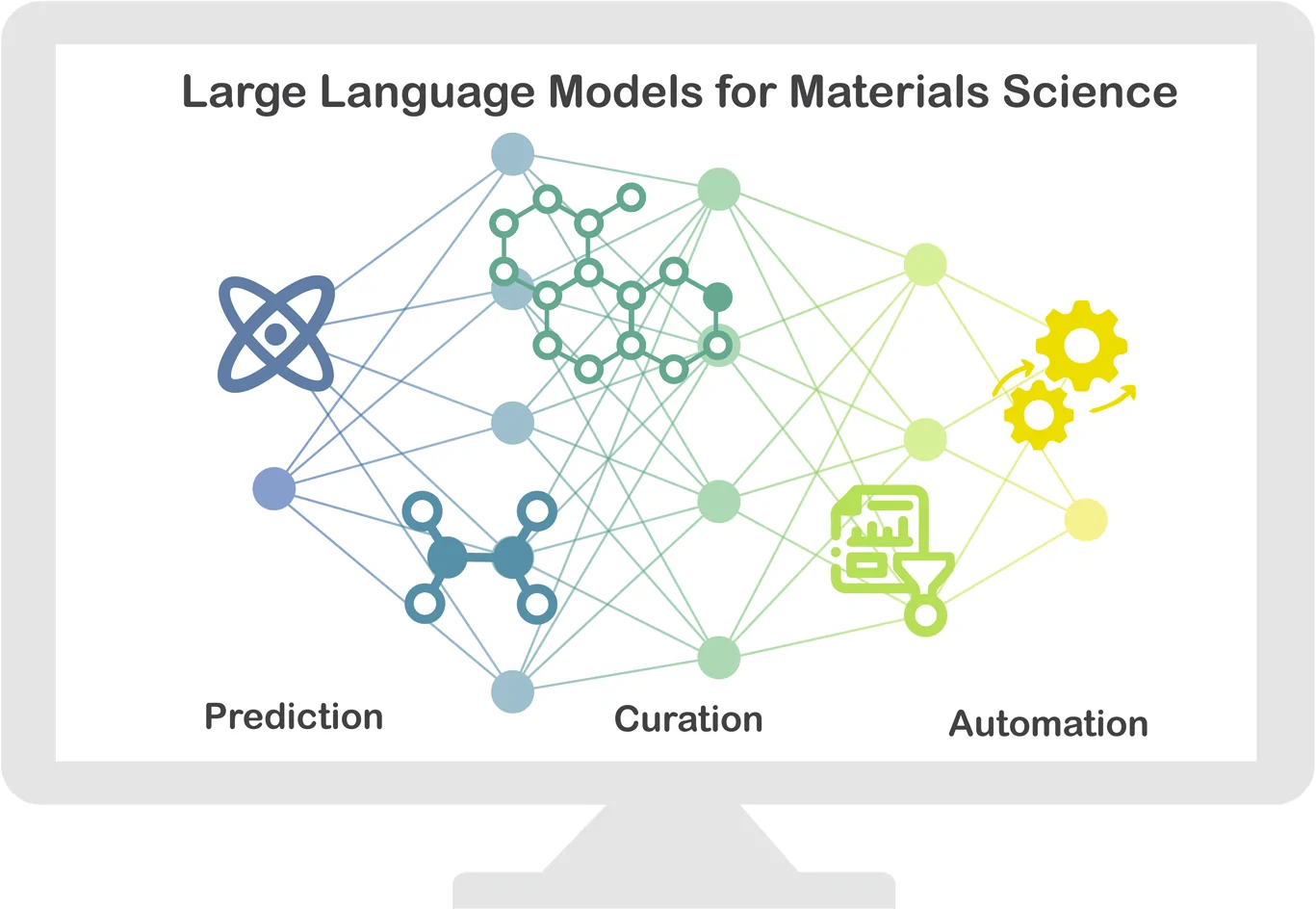

## 1 Introduction

Materials science as a discipline sits at the intersection of physics, chemistry, and often biology, and therefore requires a broad range of both skills and knowledge. A single project can cover multiple length scales, requiring various literature reviews, hypothesis generation and project planning before any experiments take place. Laboratory work can require elaborate synthesis and sample preparation routes, typically followed by a wide variety of characterization techniques. Acquired data must be processed and then analysed, either by fitting to models, compared to simulations, or calculating uncertainties. Theoreticians must understand and leverage a variety of computational techniques from density functional theory, to computational fluid dynamics, and more recently to deep learning. This may require knowing multiple programming languages, as well as having the skills to deploy code across multiple environments, like high-performance clusters or cloud services.

The rapid advancement of Artificial Intelligence (AI) – neural-network based deep-learning in particular – over the recent decade has been driven by increasingly powerful hardware and increasingly massive datasets.^1^ The culmination of this advancement is the Large Language Model (LLM), a transformer^2^ based neural network with billions of learnable parameters trained on as large a corpus of text as possible.^3^ Various LLMs exist, like OpenAI's GPT-4,^4^ Google's Gemini,^5^ Meta's LLaMA 2,^6^ and Anthropic's Claude 3.^7^ They are mostly the product of large companies with the financial and computational resources to train them, though some open source models exist.^8,9^ Despite their simple training objective of reproducing human-like text,^10^ the combination of broad training data and deep networks has resulted in impressive emergent capabilities and applicability to different domains and problems.^11^

LLMs naturally have a strong apparent understanding of the structure of natural language, being able to translate, transpose, generate, and answer questions based on texts. They are sometimes able to (or appear able to) perform reasoning and extract patterns from textual and numerical data,^12,13^ extending their use beyond just language-based applications. This combination makes them competent programmers,^14^ but also effective managers or co-ordinators in complex tasks.^15^ Whilst they perform best in workflows with a strong, LLM-independent feedback signal^16^ they are capable of automating processes in ambiguous scenarios through trial-and-error. Compared to say a Convolutional Neural Network (CNN), the transformer architecture is more amenable to multi-modality, able to combine and process encodings of text and images.^17^ This multi-modality massively expands the range of problems to which LLMs can be applied.^4,5^

Like other computer programs, but unlike human scientists, LLMs are inexhaustible – able to run all day, every day, which is useful not just in automated digital discovery workflows, but also for setups like automated laboratories or pilot lines.^18,19^ They are typically more flexible and adaptable than traditional computer programs, making them more effective when run continuously. The ability to process instructions in natural language, retrieve domain knowledge, generate code and co-ordinate systems, paired with their tireless operation and immunity to boredom make LLMs appealing tools to a materials science researcher. If used judiciously they could speed up materials discovery and perform large scale analyses previously impractical for even the largest teams of researchers.

The development of the computer revolutionised information processing and research – we argue that domain-grounded LLMs will produce another step-change in materials science. In this paper we explore the potential role of LLMs in material science, starting with a qualitative examination of the theory underpinning transformers and LLMs in Section 2. Next in Section 3 we discuss the capabilities of modern LLMs and LLM-based workflows across a variety of domains and how they might be applied to materials science. Section 4 contains two case-studies which use LLMs in materials science workflows. The first case study uses LLMs to automate tasks during 3D microstructure analysis and the second uses LLMs to extract labels for micrographs from papers using abstracts and figure captions to create a new dataset. Finally in Section 5 we examine the issues and challenges around using LLMs in research, including hallucinations, cost, and depth of understanding.

## 2 LLM theory: from attention to ChatGPT

### 2.1 Attention and transformers

**Fig. 1:**
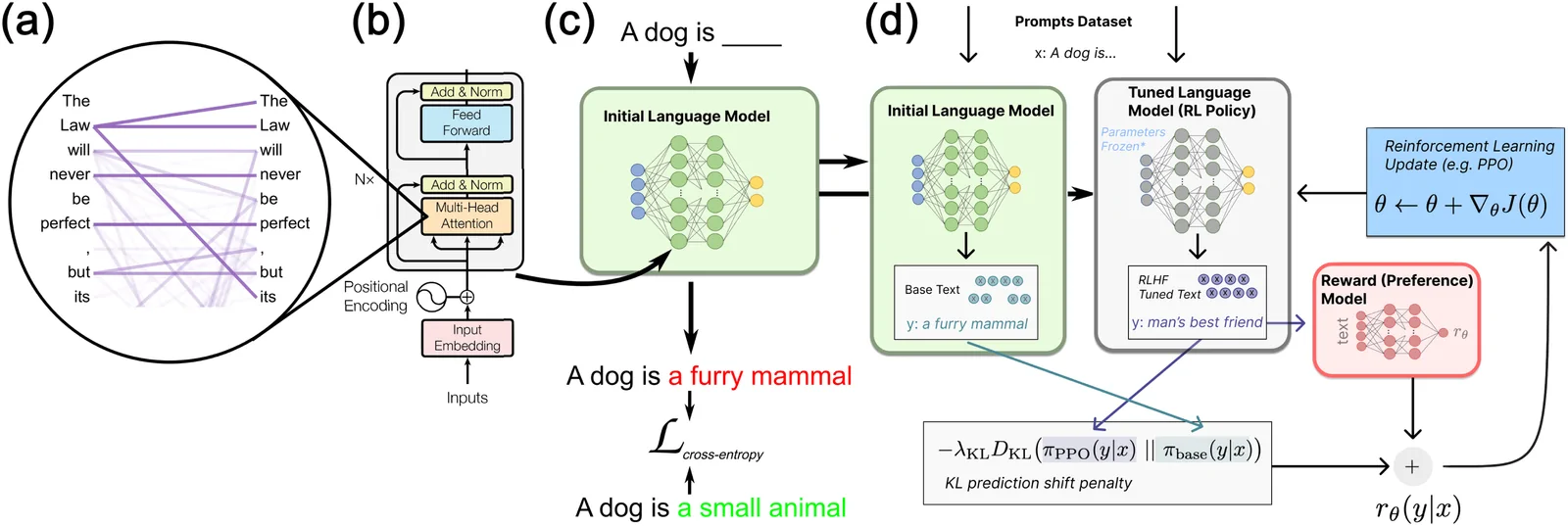
A multi-scale diagram of an LLM. (a) Shows an attention map for an example sentence, note how ‘Law’ is strongly linked to its pronoun ‘its’. (b) Shows a transformer encoder layer, made up of an attention layer and (fully-connected) feed-forward layer. Multiple of these encoder layers with associated decoder layers form an LLM in (c), which is pretrained in an self-supervised manner on a large text corpus. This LLM is fine-tuned to ensure its responses better match human preferences without diverging too much from the original model *via* RLHF, as shown in (d). Figures (a and b) adapted from ref. 2 and (c and d) adapted from ref. 20.

Attention (or self-attention), originally used for sequence modelling in recurrent neural networks,^21^ is a mechanism designed to force a neural network to consider the rest of the elements in a sequence (the ‘context’) in its representation of the current element. For a sentence that is represented as a sequence of tokens (efficient vector representations of words in terms of common sub-parts like prefixes) like “the dog chased its own tail”, attention would place emphasis on the “dog” token in its representation of the “its” token – the consideration of context allow it to model noun-pronoun relations. An example attention map for a sentence is shown in Fig. 1. A more thorough description is available in the ESI in Section S1.1.†

Transformers were introduced by Vaswani *et al.* in 2017^2^ as a neural network architecture that only used self-attention for sequence modelling. The removal of recurrent layers meant less sequential operations were needed, meaning training could be parallelized even for a single training example (like a long sentence). The use of attention in place of convolutions meant shorter distances for information propagation across a sequence, making it easier to learn long-range connections.

Despite being the most efficient way to include the whole context of a sequence of *n* tokens in a single layer, computing the interaction of every token with every other token means attention is an *O*(*n*^2^) operation. This limits the total ‘context length’ of the input sequence based on the amount of (GPU) memory. The quadratic scaling is the major downside of transformers and researchers are looking to mitigate this with techniques like windowed attention^22^ or moving to linear state-space models like Mamba,^23^ though these approaches lose global context.

Another consequence of attention is that there is no implicit ordering of tokens in the network – this information must be added in the form of a ‘positional embedding’ to the vector representation of each token in the sequence. The simplest way of doing this is word-order, *i.e.*, which number the token is in the sequence, though other embeddings like sinusoidal or learned embeddings are also used.^24^ An embedding is just a vector representation of a quantity in a new subspace – this can be as simple as one-hot encoding showing the presence of a feature or as complicated as a set of features learned by a deep CNN.

### 2.2 Pretraining and language modelling

Supervised training is updating the weights of a neural network to minimize the loss between the labels predicted by a model, *ŷ*, and the labels from the dataset *y* for a given input *x*. As an example, the *x* could be a photo of a dog and *y* could be a label from a human saying “dog”. In training, the human labels *y* are replaced with some transformation of the input *y* = *f*(*x*).

Ideally during self-supervised training the network learns strong representations of the data and can be fine-tuned or paired with another network on labelled data for specific tasks. This has two advantages – firstly that it reduces the amount of human labour needed to label the inputs, *x*, and secondly that it is believed to produce more robust representations^25^ than supervised learning, due to the lack of ‘shortcuts’ available. An example of a ‘shortcut’ is learning to predict a dog by detecting a lead, or detecting a polar bear based on ice in the background – learning these might mean ignoring more relevant and generalisable features.^26^

Transformers are parallelizable so scale well with added data and compute, and can easily learn long-range connections.^2^ Self-supervised learning requires little or no human input – massive text datasets can be collected through automated web-scraping^27^ – and generates strong learned representations. This combination makes transformers prime candidates for self-supervised learning on large text datasets to create multi-purpose language models.

One of the first works to apply self-supervised learning to large text datasets with transformers was Radford *et al.* in 2018,^10^ where a transformer was pre-trained on 7000 unpublished books before being fine-tuned on tasks like question-answering and classification. It was pre-trained using next-token prediction and operated autoregressively, *i.e.* it predicted next-token probabilities for all tokens in its vocabulary, selected the highest one, added it to the input and predicted the new next token. This was called “generative pre-training”, and the model was called a “Generative Pretrained Transformer” (GPT).

GPT's pre-training was left-to-right causal language modelling where the sequence had to be masked to prevent the transformer seeing future tokens (specifically the current token of interest) and predicting that. An alternative approach is masked language modelling, where only the current token of interest in the sequence is masked and the rest is part of the context – this is bidirectional and future context can be considered. This was the approach used for Google's BERT in 2018.^28^ The bidirectional language modelling meant BERT had higher performance on benchmarks but meant it could not be autoregressive/generative – a key factor in ChatGPT's later popularity.

### 2.3 Aligning outputs *via* RLHF

Always selecting the most probable token during autoregression leads to coherent and deterministic results, but can limit the ability of the model to be ‘creative’. Picking tokens in proportion to their probability is a simple strategy for more diverse text, and a common way to parameterise the distribution and therefore control text generation is ‘temperature’.^29^ Temperature is a scalar term introduced before the softmax function that generates per-token probabilities, with a large temperature making the distribution more uniform and thus increasing the probability of previously rare tokens.^30,31^ A low temperature does the opposite, and a temperature of ‘0’ is used to refer to most likely token selection.

‘Prompting’ is a consequence of the autoregressive learning objective of LLMs – a user's prompt is given to the LLM as a sequence and the LLM generates the most likely subsequent tokens. The model must be fine-tuned to act in a true question/answer or chatbot style.^3^ The notion of prompting has found success in other domains, like Meta's promptable ‘Segment Anything Model’.^32^

In 2022 OpenAI published a paper on “InstructGPT”,^33^ a pre-trained model which was then trained on a dataset of prompts to desired responses and finally fine-tuned *via* Reinforcement Learning From Human Feedback (RLHF).^34^ RLHF, shown in Fig. 1, contains two LLMs – a frozen LLM and the LLM to fine-tune. A prompt is fed to both, and the fine-tuned LLM's response is fed to a reward model (a NN trained to emulate human preferences) to generate a reward score. A second term is added to the reward based on the KL divergence between the frozen and fine-tuned LLM to prevent model drift. This reward is fed into a reinforcement learning policy, like Proximal Policy Optimization (PPO)^35^ to update the weights.

InstructGPT had significantly fewer parameters than GPT-3 but outperformed it, signalling the power of reinforcement learning in aligning a model's outputs with human preferences. Despite this impressive performance it is worth noting that at no point during the pre-training, training or fine-tuning are models explicitly trained to minimize factual errors or to reason – saying the sky is green goes against human preference and would therefore be penalised, but most labellers would be unaware if the model had confused ferro- and ferrimagnets if the text was otherwise coherent.

## 3 Capabilities of LLMs in research

Machine learning has seen widespread application in materials science, from characterization^36,37^ to property prediction,^38–40^ to materials discovery and design.^41–43^ They have mostly been applied in well-structured tasks with strong supervision (*i.e.*, fully labelled single-task datasets).^44^ Frequently this has involved researchers developing models trained only on their data for their problems, causing poor generalisation to new materials or processing conditions.^45^

**Fig. 2:**
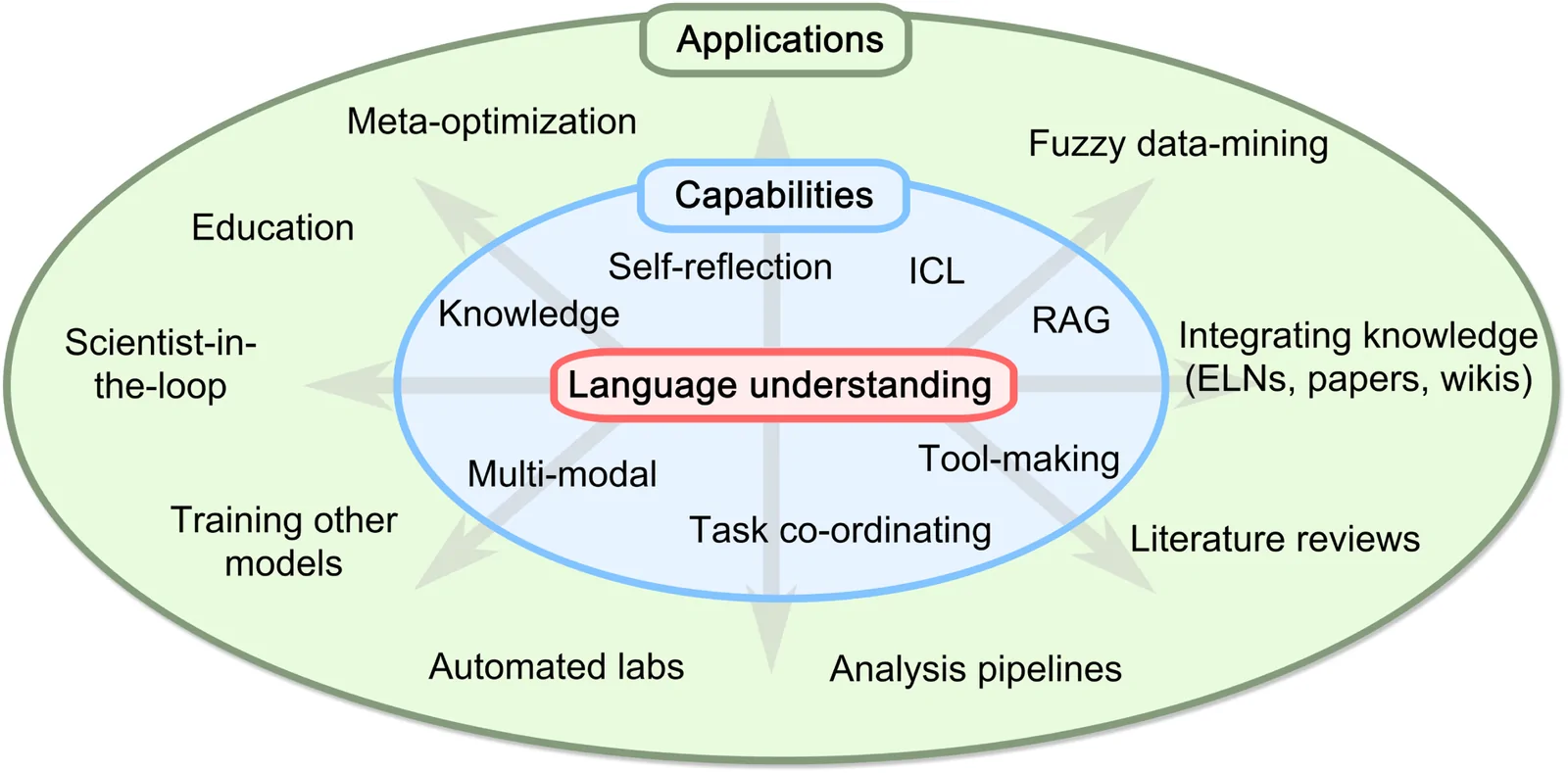
Diagram of LLM capabilities explored in Section 3 and potential materials-science related applications. These emergent capabilities can be combined with each other and integrated into traditional pipelines (genetic algorithms, databases, *etc.*) to form the different applications.

LLMs, by virtue of their large number of parameters and the scale of their training data, have strong natural language skills and emergent properties that make them promising candidates for processing more unstructured and varied data.^44^ In this section we explore some of these emergent capabilities, examine how researchers have used them in various disciplines and consider them in a materials science context (Fig. 2).

### 3.1 LLM properties: intrinsic and emergent

#### 3.1.1 Optimizing responses with prompt engineering

Prompt engineering refers to optimizing the prompt the user gives to an LLM in order to produce a ‘better’ answer or response. This can involve making the response more or less precise, conform to specific rules or schema changing the level of the explanation, *i.e.*, “explain like I'm five years old…“. Choosing the correct prompt is more of an art than a science, but some work has been done on creating and testing various principles^46,47^ or using an LLM to optimise prompts for another LLM.^48^ It has been found that it is generally better to be precise, structured (using paragraphs, whitespace and question/answer blocks) and explain to the LLM what ‘role’ it should act in.

**Fig. 3:**
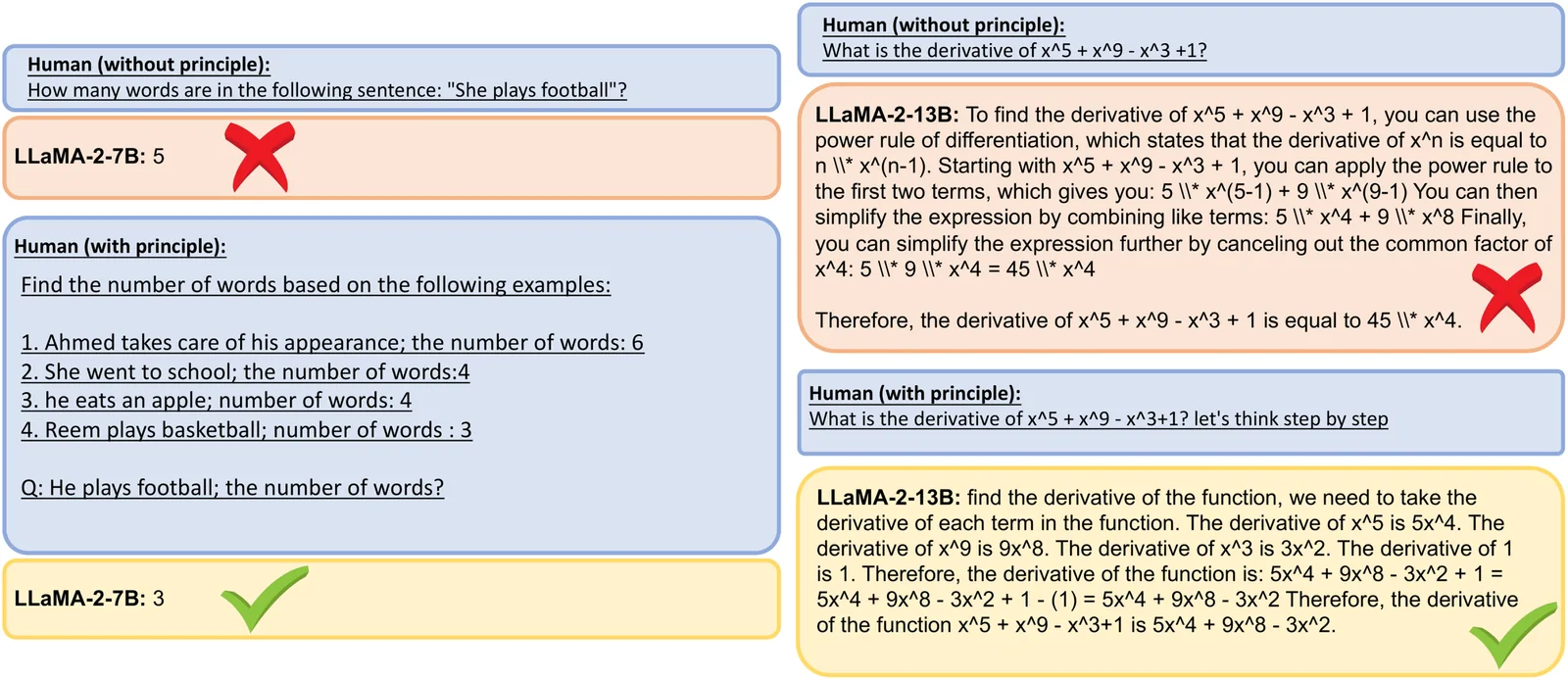
Results of applying two prompt engineering principles (giving examples and including the phrase ‘let's think step-by-step’) using LLaMa-2. Taken from ref. 47.

Adding a simple role like “You are an expert materials scientist” to the start of the prompt has been shown to improve performance on domain-specific tasks.^46,47^ Another example prompt engineering technique is output priming, where the end of the user's prompt is the start of the answer they wish from the LLM, for example “Write me a fairytale about a woodland mouse. Once upon a time…”;^47^ this plays into the LLM's most likely next token generation. Further examples of prompt engineering are show in Fig. 3.

Other prompt engineering techniques like in-context learning and chain-of-thought reasoning are explained in following sections. Example prompts for our case studies, which utilise these principles, are in ESI Sections S2 and S3.5.†

#### 3.1.2 In-context learning (ICL) and property prediction

One important emergent property that is useful to optimize a prompt is in-context learning, or few-shot learning,^3^ where a few example input/output pairs are provided to the model in the prompt. This is useful for familiarising the model with unknown concepts. Why it works is still a topic of debate, and it is a counter-intuitive phenomenon. Min *et al.*^49^ found that during in-context learning, randomly swapping output labels in examples (*i.e.* introducing wrong information into the context) only slightly decreased the accuracy of LLMs when predicting related new samples. This suggested showing the model the structure and input/output distributions were more important than demonstrating the underlying logical mapping.

However, subsequent research^12^ studied this effect with larger LLMs and found that as the number of model parameters increased, the adverse effects of wrong information in examples became more pronounced. As the percentage of swapped labels increased, the accuracy on unswapped examples dropped below 50% (the random baseline), implying these larger LLMs learnt the reverse logical mapping from the (swapped) in-context examples, rather than just the problem structure.

Google's Gemini 1.5 Pro^50^ recently demonstrated its advanced in-context learning capabilities by accurately translating English into Kalamang, a language spoken by fewer than 200 people. Initially, the model struggled with translations due to a lack of Kalamang training data. However, after processing 250k tokens of linguistic documentation on Kalamang without undergoing a traditional training regimen, it achieved near-human levels of translation accuracy.

As well as various natural language and mathematical problems,^13^ ICL has been used for both quantitative and qualitative material property prediction.^44,51–54^ However, Microsoft AI4Research noted that despite good qualitative predictions, the quantitative predictions of LLMs were lacking.^54^ Quantitative reasoning often involves complex mathematical concepts, calculations, and problem-solving. LLMs may struggle with these tasks, as their reliance on statistical patterns can lead to incorrect answers, particularly when the problems are intricate. We further discuss these problems in Section 5.

#### 3.1.3 Error correction *via* chain-of-thought (CoT) reasoning & self-reflection

Chain-of-Thought (CoT) prompting is a technique that improves the reasoning capabilities of LLMs by breaking down complex problems into sequential, logical steps.^55^ For instance, if prompted with “Anna has 15 oranges. She buys two more bags, each containing 8 oranges. How many oranges does Anna have now?”, the LLM might generate an incorrect number like “23”. With a CoT prompt like “How many oranges does Anna have originally? How many oranges does she buy? How many oranges does she have now?”, the model outputs a different, lengthier response: “starting with 15 oranges, adding 16 from two bags (8 each), and summing to 31”. CoT has been found to improve performance on tasks that require complex reasoning.^55,56^

Various explanations for this improved performance have been suggested, including that requesting the model think step-by-step increases the length of the sequence. Recall that during autoregression the whole sequence including the output so far is fed into the model to generate the next token – adding more tokens gives the model more context and thus more ‘space’ to compute with, as more text means more interactions in the attention layer. Another possibility is that longer sequences reduces the space of likely sequences to those that contain the correct answer; if the model has repeated “John has 4 apples” multiple times as part of its explanation the probability of outputting future tokens that use (directly or indirectly) a different number of apples is reduced compared to directly outputting the answer.

Self-reflection is a consequence of ICL and CoT and involves giving an LLM an evaluation of its previous prompt in a new prompt, this can be pointing out errors or a broader evaluation. This can be from a human,^55^ a program (*i.e.*, a stack trace),^57^ another LLM^58^ or even itself.^59^ Self-reflection improves performance, potentially for the same reasons as ICL and CoT, but also because correcting a wrong output may be a simpler task than generating the correct output *de novo*.

#### 3.1.4 Pre-existing and fine-tuned materials domain knowledge

LLMs are trained on large corpuses of text that contain facts about the world, including large datasets of scientific papers.^60^ Common facts will be repeated many times across these texts, making it statistically likely that an LLM will reproduce them when prompted to. Microsoft AI4Science found that “In biology and materials design, GPT-4 possesses extensive domain knowledge”, which they evaluated by asking domain experts to rate outputs about various drug molecules, general materials design principles, mathematical concepts like PDEs and more.^54^

The ability to act as an oracle for common shallow information across many domains^61^ is useful in a multi-disciplinary field like materials science, but the model generalization encouraged by the pre-training task and autoregression can limit the usefulness of LLMs for deep information recall.

One way of overcoming this is fine-tuning on domain-specific knowledge. This domain specific knowledge can be collected by traditional web-scraping or through using ML models^62^ and then used to fine-tune a language model like BERT.^28^ Models like SciBERT^63^ outperformed BERT and other state-of-the-art models for tasks like text classification or Named Entity Recognition (NER). MatSciBERT^64^ took this process a step further and fine-tuned SciBERT on materials science specific data to outperform SciBERT on materials science text tasks. Jablonka *et al.*^65^ also demonstrated that fine-tuning GPT-3 on chemistry and materials science tasks allows the model to achieve high performance for property prediction, often surpassing traditional machine learning models, especially in low-data scenarios.

Full fine-tuning of any large ML model is expensive and risks ‘catastrophic forgetting’,^66^ where a model loses information from its general (pre)training during the domain-specific fine-tuning. One way to alleviate both the cost and catastrophic forgetting problem is Parameter-Efficient-Fine-Tuning (PEFT), where only a small subset of the model's parameters are updated. Example PEFT schemes include LORA,^67^ adapters^68^ and prompt/prefix-tuning methods.^69,70^

Textual representations of molecules like SMILES^71^ and SELFIES^72^ have seen success in transformer networks for property prediction^73,74^ and molecular generation.^75^ Work has been done on fine-tuning LLMs to include these textual representations, either by training exclusively on these representations or including them in-context in training data, showing promising results in molecular generation, inverse design, and property prediction.^76–78^ Similar studies have been done for crystal structure prediction and generation, for example by fine-tuning an LLM on millions of .cif files^79^ or on custom string representations.^80^

#### 3.1.5 Comprehensive programming skills

Large quantities of text exist online (and therefore in LLM training sets) about programming: discussions, help forums and source code, and the move towards approachable, high-level programming languages like Python means source code is increasingly similar to natural language. These two facts mean LLMs are proficient at generating, modifying, correcting and summarizing code in a variety of languages for a variety of tasks.^14,81^

Programming is ubiquitous in modern science, from data processing, analysis, visualization, simulations, instrument interfaces, *etc.* and the ability to write reasonable code across all these different tasks is obviously useful for researchers. LLMs have been shown to be proficient in these tasks in a materials science context.^54^ The ability to code in different contexts is also fundamental for many of the workflows explored in Section 3.2.

#### 3.1.6 Multi-modality – enriching materials characterization

The tokenization, positional embedding and attention mechanics of transformers are highly flexible and therefore capable of jointly modelling different modalities and tasks.^82^ A prominent example of this is OpenAI's CLIP (Contrastive Language–Image Pre-training),^17^ where a model is trained to maximize the similarity of text and image representations for text-image pairs collected from the internet. The success of CLIP and other multimodal representations^83^ has led to the rise of Vision Language Models (VLMs) like GPT-4,^4^ LLaVa^84^ and Gemini^5^ which can use information from text and images to aid in the generation and processing of both.

Joint text-image reasoning has the potential to be a useful analysis tool when combined with existing datasets of materials images and descriptions – consider searching the literature for microstructures that display similar features, defects or artefacts to your data, with potential answers from related papers signposted.

Images are not the only mode of data that transformers can learn to use with text. There are examples using videos *via* 3D CNN embeddings,^85^ speech/audio using spectrograms^86^ and even graphs *via* Graph Neural Network (GNN) embeddings.^87^ Notably, OpenAI's Sora^88^ has extended this versatility further by generating high-fidelity videos, demonstrating the application of transformers beyond static images to dynamic, temporal data.

Finding suitable embeddings for the wide range of characterization techniques that exists in materials science (CLIP for micrographs, GNNs for crystallographic information from XRD, 1D CNNs/LTSMs for spectral data) and fine-tuning a transformer or LLM with them could be a promising direction for injecting domain-specific knowledge or priors. For example, embeddings for spectroscopic data could be extracted from the hidden layers of CNNs that have shown good performance in material-specific classification or quantification tasks in Raman^89^ or P/NMR^90,91^ spectroscopy, though a larger, more varied training dataset would be needed to ensure they generalise well.

Multi-modality has become an increasingly important focus of LLMs,^50,92^ with recent examples like GPT-4o being ‘natively multi-modal’,^93^ meaning they are trained to input and output tokens of various modalities (rather than, say, generating an image by producing text to describe the image and feeding that into a different text-to-image model). The success of a multi-modal materials science LLM will depend on the quantity and variety of data from the various modalities, as well as how amenable the modalities are to tokenization.

### 3.2 Resulting workflows

These properties are flexible and composable, meaning they can be combined in a wide range of potential workflows in various domains, including materials science.^44^ Below are a few examples of such workflows, and though they are split into separate sections there are strong links and similarities between them. The key commonality is letting LLMs act as high-level managers whilst other, more robust systems perform low-level tasks.

#### 3.2.1 RAG: generation from custom datasets

Retrieval Augmented Generation (RAG) involves performing a lookup into a traditional database and using the retrieved information as part of a prompt to an LLM in order to achieve better or more accurate generation.^94^ The lookup is usually based on some function of a user request – one common way to match the semantics of a user's search to a database is ‘vector search’, where embeddings of every item in a database are pre-computed using a language model like BERT^28^ and the ones with the highest similarity (usually cosine similarity) with the embedding of the user's request are returned.^95^

RAG has several benefits to LLM workflows:^96^ firstly, hallucinations are reduced as models only need to process existing information in a prompt rather than generate (or fabricate) it. It is more interpretable as the retrieved documents can be linked back to confirm the results. Finally, these databases can be updated simply by computing the embeddings for the new items – without RAG the LLM would need to be retrained or fine-tuned to add the new information.

The utility afforded by RAG is clear – many companies are trying to use or sell it as a service,^95^ and it is a feature in GPT-4.^4^ It is not hard to see how LLMs paired with a vector database of materials papers using, say, MatSciBERT's^64^ text embeddings could prove useful in research. Indeed, some have already used RAG alongside knowledge graphs for materials design.^97^

#### 3.2.2 Tool-using and making for analysis pipelines

LLMs can be trained to use tools like search engines,^98^ translations, mathematics plugins, *etc.*, which is useful in situations where they typically underperform, like arithmetic.^99^ This can be achieved through ICL and ‘prompt managers’ by providing details of the tools and situations in which to use them to the LLM and running the generated code or API (Application–Programmer Interface) calls.^100,101^

Another more involved approach used by Toolformer^99^ was to use ICL to make LLMs annotate an existing language dataset with API calls for a variety of tools where it deemed them useful. They then fine-tuned the model on that data, including a loss term to indicate when the API call improved the accuracy of the generation. This approach has the benefit of not relying on prompts, which can crowd the limited context window and sometimes be ignored by the LLM.

LLMs can generate code and as such are able to produce their own tools. The LLMs As Tool Makers (LATM)^102^ framework used LLMs to generate tools which can then be used by other LLMs. They noted that tools were harder to make than use, so had a more powerful LLM (GPT-4) generate the tools, tests and documentation and a weaker LLM (GPT-3.5) use the tools.

A tool-making and using LLM with a human-in-the-loop could be useful for materials science problems where the workflows and requirements are varied (in terms of data types, desired analyses or post-processing) like in image processing. This could be further combined with RAG on relevant papers for domain knowledge engagement and a database of generated tools to obviate the prompt context window limit. Progress has been made on that front, including ChatGPT integration for the ImageJ macro language inside ImageJ itself.^103^

#### 3.2.3 Task integration: the future of automated labwork?

Various papers have shown that LLMs can act effectively as managers of various sub-components, like other software tools or even other LLMs (called ‘agents’). This tends to involve feeding the outputs of these tools or LLMs as a prompt into the manager LLM.

One fun example of co-ordination is ‘Generative Agents: Interactive Simulacra of Human Behavior’,^104^ where LLMs acted as villagers in a sandbox with a set of possible actions and locations. They performed inter-agent communication and had a recursively summarised memory of events fed into their prompt to maintain consistency.

Maintaining a memory external to the LLM (*i.e.*, in a text file) has been explored by studies like MemGPT^105^ which aimed to emulate modern operating system memory management to allow LLMs to perform tasks like large document summarization and multi-session chats. To achieve this they had a traditional scheduler with events for document uploads and timers, and allowed the LLM a set of functions to call in response including send messages, read, write, and send interrupts.

‘Coscientist’^18^ used LLMs as a coordinator to design, plan and execute chemical research. It can call web search APIs, generate and execute Python code, search documentation, interact with and write code for physical hardware. Despite the need for manual intervention to execute the experiment, it is a promising example of how LLMs can orchestrate various research and lab tasks. Similarly, ‘ChemCrow’^106^ is an LLM chemistry agent designed to tackle tasks in organic synthesis, drug discovery, and materials design. The agent uses an iterative, automated chain-of-thought reasoning approach to plan its approach which it executes *via* a set of prewritten tools. Like ‘Coscientist’, it has access to web-search, can write its own code and interface with a robotics lab, but it also has a variety of molecular, reaction and safety tools it can employ.

Much effort is being made to integrate LLMs with robotics^107^ as task planners,^108^ reasoning agents^109^ or as part of a broader vision-language-action multimodal model.^110^ Advancements in grounded robotics and embodied AI will further development of automated labwork, improving all-in-one workflows like Coscientist. However, it is worth noting the margin for error (and hallucinations) is much smaller in labs, where a wide variety of hazardous chemicals and processes are handled frequently.

#### 3.2.4 Optimization loops and ‘flow engineering’

Another useful LLM-based workflow is meta-optimization, where instead of generating an optimal solution to a problem, an LLM generates the code to produce the optimal solution. ‘Eureka’^111^ used an LLM to generate reward functions for reinforcement learning applied to robotics simulations. They used a genetic algorithm, where the best generated reward functions and their summary statistics were included in a prompt to allow the LLM to ‘reflect’ and then synthesise a new, better set of reward functions. The framework outperformed expert-written reward functions on a large majority of tasks.

**Fig. 4:**
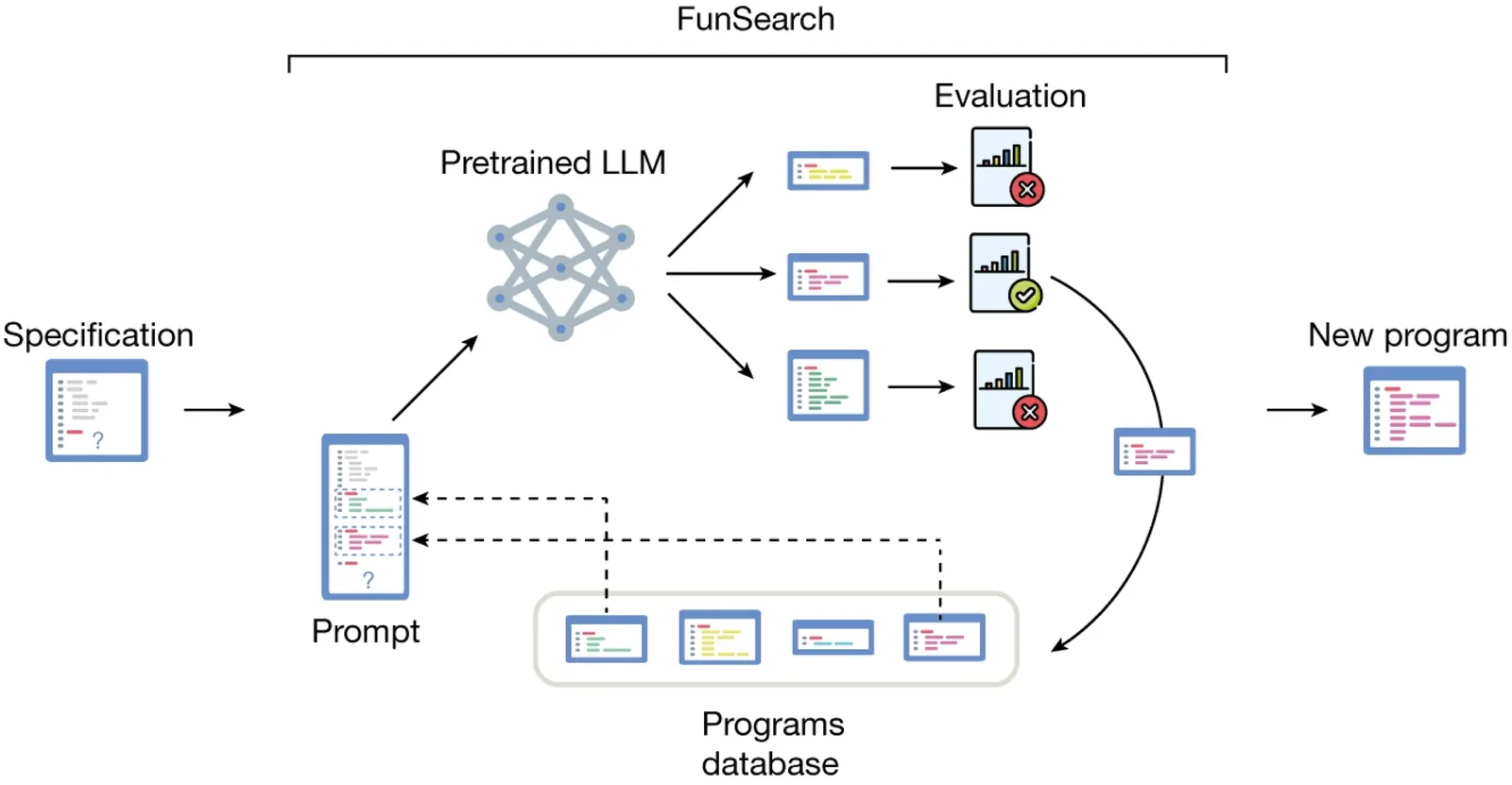
Diagram of the FunSearch^16^ evolutionary workflow, where an LLM is prompted with a problem specification and best example heuristics from the previous iteration and tasked with combining them to generate better candidate heuristics to solve a problem. These new heuristics are evaluated, stored in database and the process repeated. This process was able to discover a new upper bound for the largest cap set in 8 dimensions. Taken from ref. 16.

DeepMind's FunSearch^16^ followed a similar approach, using LLMs to generate heuristics for approximating solutions to mathematical problems like the cap set or online bin packing problem (Fig. 4). They also used a genetic algorithm framework, asking the LLM to combine aspects of best-performing heuristic programs to generate new ones. Like Eureka, this relied on a combination of ICL, CoT and a feedback signal from an external program – in Eureka's case this was RL simulations using the reward functions which tracked quantities like time upright and for FunSearch this was small validation programs which evaluated how well the heuristic performed (*i.e.*, if the cap set was valid and how large it was).

The FunSearch process found a new upper bound for the largest cap set in 8 dimensions, exceeding previous upper bounds found by human mathematicians. Despite this success, this was not a triumph of artificial mathematical understanding – a review of FunSearch noted it was “remarkable for the shallowness of the mathematical understanding that it seems to exhibit”^112^ – instead it was proof of the power of LLMs inside an evolutionary framework.

The LLM in FunSearch did not need to always be correct – the strong feedback signal from the deterministic evaluators ensured mathematical correctness. This is therefore a good model for reconciling the LLM's occasional hallucinations with the need for scientific accuracy. Based on the results, it seems the key contribution of the LLM was to reduce the search space of the genetic algorithm from all possible functions to all plausible functions, hugely increasing convergence time and final performance.

A recent meta-optimization coding paper is AlphaCodium,^113^ which used a multi-step framework combining reflection on a given specification, human-written tests and LLM-generated tests. The emphasis on tests was because they noticed it was easier to generate useful unit tests (which could then improve future generation) than the correct code. They called this process ‘flow-engineering’ and improved pass accuracy on challenging code problems from 19% with just GPT-4 to 44% with GPT-4 as part of the AlphaCodium flow. A useful feature of all these meta-optimization loops is that they tend to be LLM-agnostic, *i.e.*, GPT-4 would work equally as well as LLAMA or FALCON.

An important aspect of FunSearch (and other LLM meta-optimizations) was that the programs it generated were interpretable by humans. By examination of the program that generated the new upper bound, the researchers found a new symmetry in the cap set problem. This human-in-the-loop approach to optimization and discovery is appealing in the natural sciences – one could imagine tasking an LLM evolutionary framework to find new functionals in DFT or approximating solutions to physically-relevant combinatorial problems like the max-cut problem.^114^

## 4 LLM workflows in materials science: two case studies

### 4.1 Case study 1: automated 3D microstructure analysis

In Section 3.2 we examined the potential of LLMs to make, use, and orchestrate various tools into automated workflows. Typical materials data analysis pipelines require a combination of domain knowledge, statistical understanding, and various programming skills. The programming required is often non-trivial, involving data handling, conversion, simulations, plotting, *etc.*

LLMs have the potential to reduce the knowledge and skills barriers for these workflows, by offering a natural–language interface to a wide pool of programming knowledge, tool co-ordination, and automation.

**Fig. 5:**
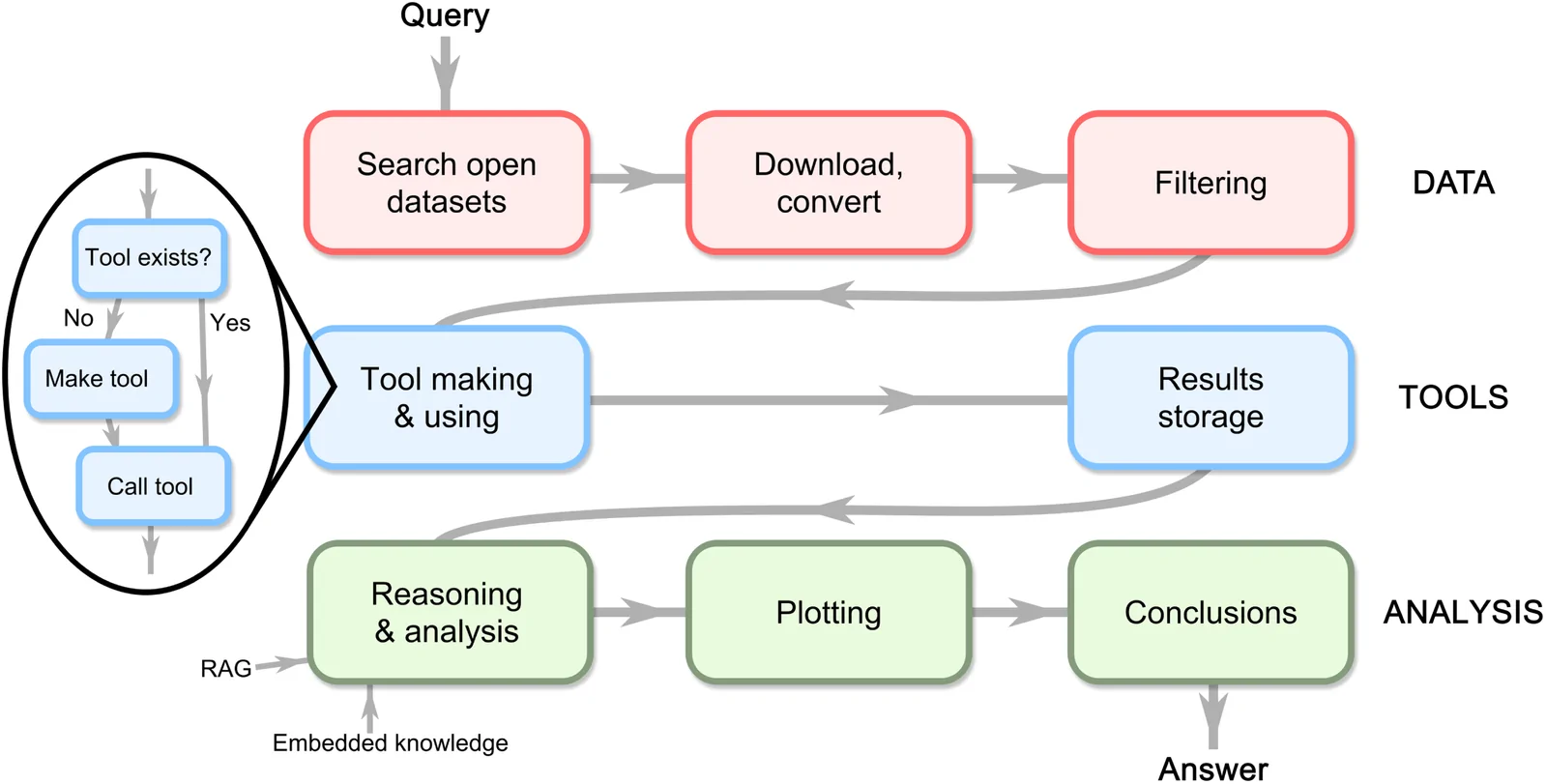
Diagram of MicroGPT's workflow, beginning with dataset collection and filtering. This is followed by tool making and using to extract metrics from the data – this can be an existing tool from its toolkit like tortuosity calculations or created for the specific query.

As an example, we developed “MicroGPT” – a specialized chat-bot to streamline 3D microstructure analysis. Prior work has been done on LLM-guided materials design, like Text2Concrete^44,115^ or BatteryGPT,^116^ where the former employed an iterative generator/critic approach and the latter extracted relevant manufacturing parameters and knowledge from specialised databases. We focused on integrating simulation and analysis tools into the design process (Fig. 5). MicroGPT has a variety of functionalities:

• Data acquisition: MicroGPT can conduct searches for open-source datasets on Zenodo (an interdisciplinary open-access repository) and employ functions to download these datasets using the links available on the respective web pages.

• Filtering: it can retrieve the dataset's metadata, parse it and subsequently refine the data according to the user's (natural language) specifications. Finally, it can organize the filtered data into a newly created file directory.

• Integrated simulations: it can apply simulation tools to the 3D microstructures, documenting the simulation outcomes in formats such as .csv. These results can then be automatically uploaded to a cloud provider given an API key.

• Data analysis: it can compare various datasets, collect simulation results and based on user requirements, formulate hypotheses, and provide recommendations.

• Data visualization: the results of the data analysis can be plotted, either as histograms for distributions of single properties across the dataset or scatter plots to examine the correlations between properties.

• Tool making and reuse: custom tools can be developed based on the user's specifications, stored and reused in later analyses. Over time this will lead to a library of useful and relevant functions that extend MicroGPT's capabilities.

This was achieved using GPT-4's API. Custom functions were defined in terms of their description, arguments and return values (in .json format) and input to the GPT using OpenAI's ‘function calling’ so the LLM would call them when appropriate. These were implemented in Python and run client-side. We added more system prompts with explicit instructions to improve stability (see Section 3.1 and ESI Section 2†).

To demonstrate these functionalities we used MicroGPT to collect and filter data from “MicroLib”,^117^ a collection of plausible, synthetic 3D microstructures generated from DoITPoMS^118^*via* SliceGAN.^119^ It then filtered the structures to only ones related to materials with specific characteristics. Relevant 3D metrics like tortuosity, effective diffusivity, volume fraction, and surface area were calculated using TauFactor 2(ref. 120) *via* a function call.

MicroGPT collated the results, identified a potential outlier, and suggested some materials for further investigation. It successfully correlated metrics such as tortuosity and surface area with desired properties like high flow rates and extensive surface areas for efficient performance.

MicroGPT is a promising example for LLM-assisted analysis workflows, leveraging many of the properties in Section 3 like natural language understanding, programming skills and chain-of-thought reasoning. The grounding of MicroGPT using tool like search APIs, RAG, *etc.* is a future research direction which could both reduce factual errors and enhance domain knowledge engagement for reasoning and hypothesis generation. A detailed example dialog and system prompts are available in the ESI Section S2.†

### 4.2 Case study 2: labelled microstructure dataset collection

There are few large (>1000 entries) micrograph datasets that cover a range of instruments and materials, and even fewer with material-specific labels. The Cambridge DoITPoMS^118^ library contains around 900 labelled micrographs of various materials captured mostly with optical or reflected light microscopy. Another dataset from Rosella *et al.*^121^ contains 22 000 SEM images of materials with taxonomic labels. Biological datasets are larger and better collated,^122^ contributing to the success of generalist deep-learning approaches like Cellpose.^123^

Materials science papers contain many high-quality examples of micrographs taken using a variety of techniques, usually with descriptive captions and abstracts. Traditional string-matching approaches like regex may be capable of detecting whether a given figure contains a micrograph and extracting the instrument used to take it from the caption, but detecting which material is present is generally not possible. The problem is further complicated if the figure contains multiple sub-figures like plots or diagrams alongside the micrograph, which occurs frequently.

Various approaches to automated materials science data extraction exist in the literature, using Natural Language Processing (NLP) techniques like Markov models, Conditional Random Fields (CRFs)^124^ and word embeddings from models like word2vec^125^ for tasks like Named Entity Recognition (NER).^126,127^ These NLP approaches can be combined with web-scraping to create extractor tools like ‘ChemDataExtractor’^128,129^ and ‘MatSciE’,^130^ which aim to automatically create datasets from text entities and tabular data in papers matching a given search query.

Image-based paper data extractors also exist, like ‘ImageDataExtractor’^131^ which is capable of detecting electron microscopy images from figures, identifying their scale and segmenting any nanoparticles present. ‘EXSCLAIM’^132^ uses rule-based NLP and image processing to extract images and assign hierarchical labels based on the figure caption. These extractors have found use in generating structured datasets for nanoparticles,^133^ photocatalysts^134^ and self-cleaning coatings.^135^

LLMs and VLMs offer solutions to both these problems, displaying strong natural language skills and the ability to consider wider contexts like paper abstracts and therefore enabling large-scale automated micrograph collection and labelling from the literature. Some work using GPT-4V for extracting information from a paper's figures exists, for example analysing graphs (PXRD plots, TGA curves, *etc.*) in reticular chemistry papers^136^ by treating each page in the .pdf as an image.

We began by scraping paper metadata (title, authors, abstracts, links, *etc.*) from arXiv and chemrXiv that matched the query ‘microscopy’ *via* their APIs. For each paper we then downloaded the .pdf, ran the ‘pdffigures2.0’ figure and caption extractor^137^ and saved the image–caption pairs alongside the metadata. We further extracted the subfigures for each figure by detecting connected components surrounded by whitespace and removing small (less than 200^2^ px) results.

A two step screening process was used, first we fed captions and abstract to a text-only LLM (GPT-3.5 or 4) to determine if a micrograph was present, what instrument was used and the material depicted. Next we prompted a VLM (GPT-4V) with the specific subfigure, its parent figure, caption and abstract to work out if that specific subfigure was a micrograph and again what instrument was used and what material was imaged.

**Fig. 6:**
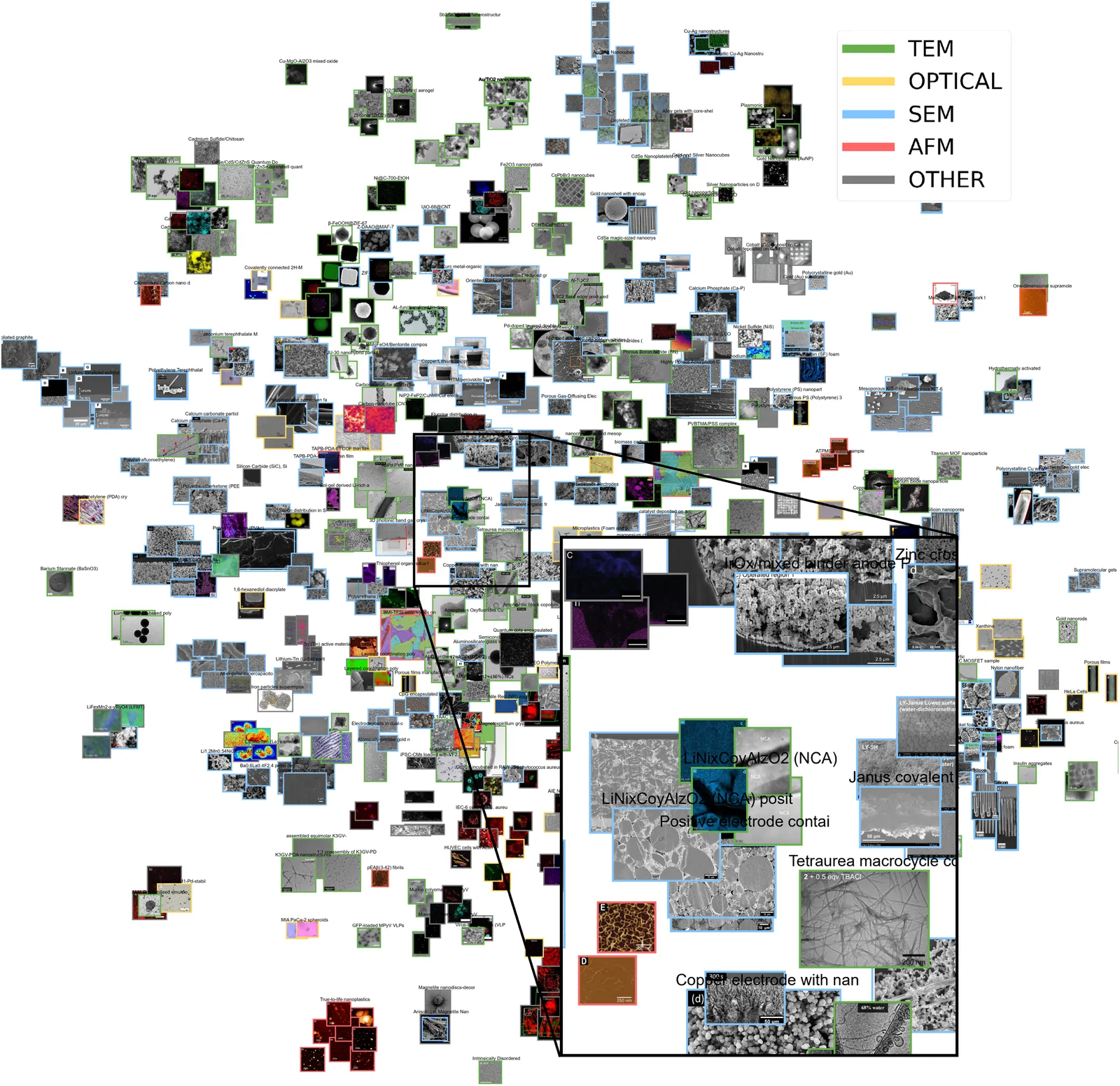
T-SNE plot of the MatSciBERT^64^ embeddings of the ‘material’ label assigned by the LLM to each micrograph in the dataset based on the paper abstract and figure caption. Border colour denotes the instrument the micrograph was taken with. Similar materials are grouped together: nanoparticles in the top right, energy materials in the middle on the left and quantum dots in the top-left corner. Best viewed zoomed in. The inset zoom displays some micrographs with similar labels in embedding space (in this case mostly energy materials) grouped together. Further examples of extracted micrographs and their generated labels can be seen in Fig. S3.†

After running this process on 382 papers (a subset of the 14 000 scraped) we collected 842 micrographs, each with an instrument and material label – a link to the dataset is available in Data availability. Fig. 6 shows a visualization of the dataset, where micrographs are grouped based on how similar the MatSciBERT^64^ embeddings of their labels are. The LLM-generated labels were compared to human labels recorded with a custom GUI (developed for this case study) for each figure and subfigure to work out the accuracy of the process.

**Fig. 7:**
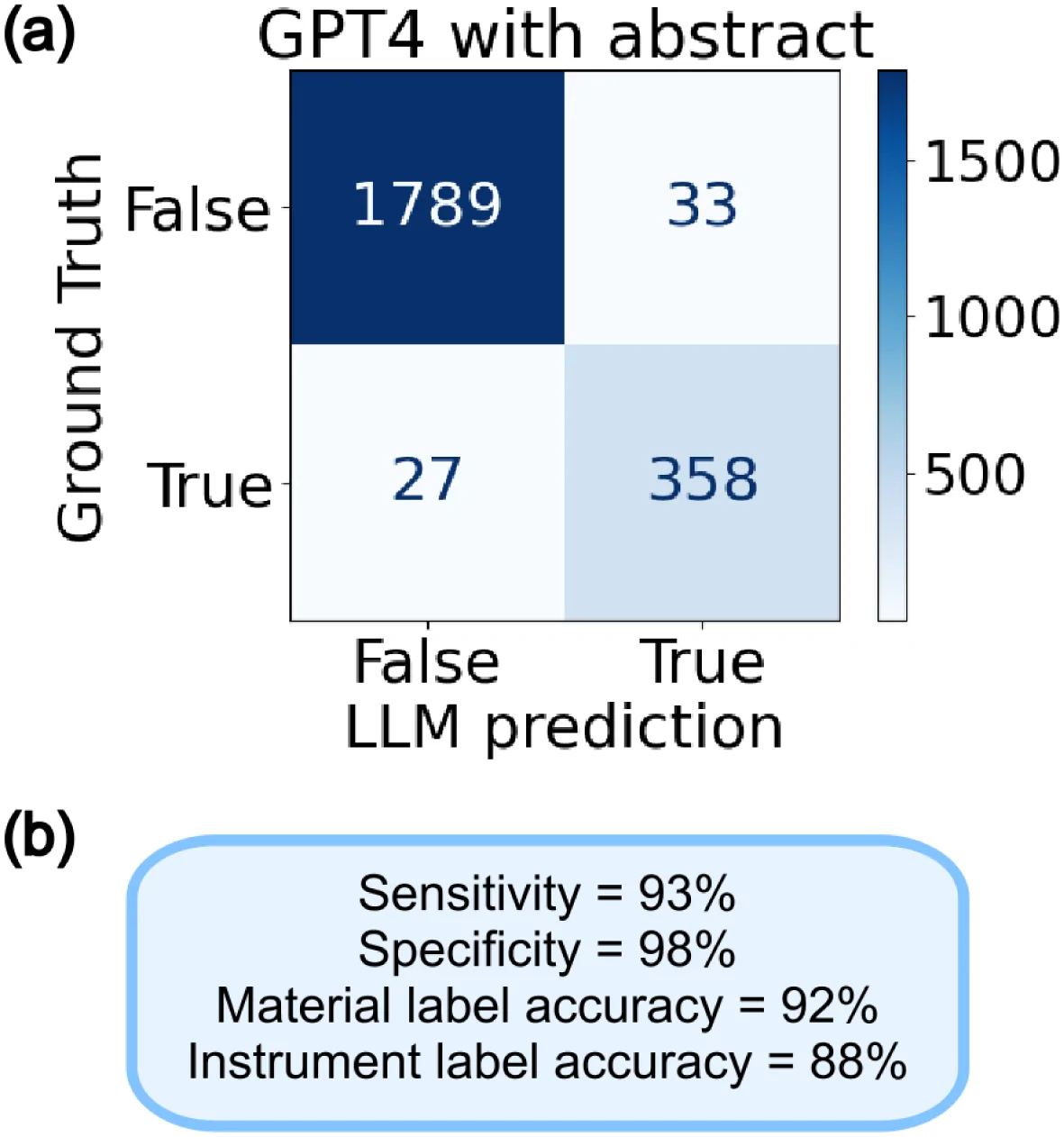
Evaluation of micrograph detection performance of GPT-4 supplied with figure caption and paper abstract, including a confusion matrix in (a) and statistics in (b). GPT-4's performance is strong across the board, with good sensitivity, specificity and accuracy for instrument and material labels.

During the case study we evaluated the performance of various setups, including using GPT-3.5 or −4 and whether we prompted the LLM with the abstract or not. GPT-4 far outperformed −3.5, and using the abstract led to a minor improvement over not. See Fig. S4 in ESI Section S3.4† for details. The performance of GPT-4 with abstract is shown in Fig. 7, with a sensitivity and specificity above 90% for micrograph detection, and material and instrument accuracy above 80%.

We found that LLMs were competent labellers, sometimes matching human labels almost exactly. The success is mostly attributable to the fact that the task could be done with no materials-science specific knowledge due to how well-structured scientific captions are. The text-only LLMs make mistakes when the caption mentions ‘image’ without showing a micrograph, *i.e.*, in a plot of statistics taken from an SEM image. The VLM did not have this problem, and there were no false positives after the second step (though this may be because the first step was already a strong filter), this is discussed further in ESI Section S3.3.† No further manual curation was applied to the dataset.

This LLM-based workflow is potentially more flexible than approaches using classical NLP in that rules and desired data can be changed more easily and it is more robust to varying caption syntax – the authors of EXSCLAIM noted shortcomings in the rules-based approach, namely that nearly a third of the extracted images did not receive “a substring of text from the caption distributor”, and that this “is where transformer-based NLP models stand to make the greatest contribution to the overall pipeline”.^132^

However, this increased flexibility comes at the cost of potential hallucinations, lack of explainability, poor reproducibility and a larger computational overhead (explored further in Section 5). A synthesis of the two approaches, for example using classical NLP to ground LLM labels, could prove fruitful. Utilities like the scalebar recognition of EXSCLAIM or ImageDataExtractor could also improve the workflow.

More details on the setup, including the system prompt, can be found in Section S3 of the ESI.† Three representative micrographs alongside a comparison between their labels and the original caption is available in Fig. S3.† The code needed to reproduce the results or run on more specific queries is available in Data availability. In the future we intend to apply this automated approach to a much wider dataset, with the hopes of creating a varied micrograph dataset for computer vision applications.

The method based on LLM and VLM to extract datasets offers high accuracy with good understanding and interpretation of complex data types like images and scientific texts. But it is computationally intensive, may provide misleading inaccurate information, and has concerns regarding explainability, transparency, and reproducibility, which will be further discussed in Section 5. While previous methods may be limited to data modality, they yield more stable results, allow users to easily trace the source of the results, and require less computational effort. Considering when and which method to use is worth thoughtful consideration.

## 5 Issues and challenges

There are naturally a few problems with integrating LLMs into materials science workflows, the most prominent and concerning being that of hallucination or confabulation. Huang *et al.*^138^ provide a taxonomy of hallucination types, separating hallucinations into two main types: factual and ‘faithfulness’. Factual hallucinations involve being wrong or fabricating facts, and ‘faithfulness’ hallucinations involve ignoring user provided instructions or information, or making logical errors.

Various causes of hallucinations have been suggested,^138^ including (but not limited to) pre-training on incorrect or duplicated data, randomness from output sampling and a ‘capability misalignment’ between the demands made by RLHF fine-tuning and the model's capabilities – LLMs may have been trained to hallucinate in some cases.

These fixes for hallucinations exist mostly at the dataset or training level, which is difficult for all but the largest research groups to manage. As noted in Section 3.2, RAG is a good way to mitigate factual hallucinations,^138^ as manipulating existing data by RAG is easier than updating knowledge recall methods, and it can supply a model with information from outside its training set. Chain-of-thought reasoning can also sometimes mitigate logical hallucinations,^138^ though asking a model to correct itself requires knowing the output was wrong in the first place, reducing the value-add of LLMs.

Microsoft Research AI4Science noted some hallucinations of GPT-4 whilst handling complex topics like materials science and chemistry. In generating silicon crystal structures, GPT-4 initially provided incorrect atomic positions, which were partially corrected after further prompts but still contained some inaccuracies. When predicting the electrical conductivity of inorganic materials, GPT-4's accuracy was only slightly better than random guessing, misclassifying several compounds. While generating code for pressure-temperature phase diagrams, GPT-4's use of simplified equations led to inaccurate phase boundaries. Additionally, in quantum chemistry discussions on symmetry and antisymmetry, GPT-4 used correct problem-solving approaches but flawed algebraic calculations, leading to erroneous conclusions (the wavefunction is antisymmetric).^54^ Hallucinations are more likely to occur when an LLM is processing data in areas where it has a knowledge gap,^139^ which is likely for broad, deep domains like materials science.

As well as contributing towards hallucinations, data duplication (alongside autoregression and the pre-training objective) can also contribute to an LLM's tendency to output towards a generic or modal answer. This is not just a problem if asking about uncommon materials or analysis techniques but also if using LLMs to explore a hypothesis space, design principles or automate experiments. The risk of using LLMs in research is that we reinforce existing biases and overlook unconventional approaches not well-represented in the training data.

Reproducibility is also a challenge for LLMs. Although they can be run deterministically with a temperature setting of 0 (see Section 2.3), this limits their ‘creativity’ and makes them less useful for generative tasks, like molecular or materials design. Temperature is not the only factor limiting reproducibility, as small changes to prompt phrasing can have large changes on the result.^140^

There are practical issues to implementing LLMs in materials research. The models are expensive to run if using a cloud provider like OpenAI's API, or if run locally require powerful GPUs with at least 8 GB of VRAM (which are currently expensive). Quantizing these models (storing their weights with less floating-point precision) can ameliorate this, at the cost of slightly diminished accuracy. For research groups or companies dealing with sensitive or proprietary data there are privacy issues around uploading data to cloud-based LLMs – running local models is a good workaround but requires more know-how.

The large parameter count of LLMs that enables their impressive language capabilities also makes them difficult to interpret, both algorithmically and practically. Sampling a single token, to say, examine the associated attention maps, requires non-negligible compute, and asking a model to explain its own outputs is also prone to hallucinations.^140^ Work is ongoing on developing explainability methods for LLMs.^140,141^

LLMs struggle with performing quantitative reasoning. Even a language model like ‘MathGLM’^142^ that was fine-tuned on large synthetic datasets of arithmetic problems struggled to achieve high accuracy on multiplication problems. Its accuracy decreased as the number of digits increased, leading some to suggest^143^ it (and LLMs in general) had not learnt the underlying rules of multiplication. The most promising avenue for improving this seems to be teaching the LLM when to use an external tool like a calculator (see Section 3.2).

## 6 Conclusion

To conclude, we have explored the basic theory behind LLMs, linking their industrial-scale self-supervised pre-training and reinforcement learning-based fine-tuning to their impressive natural language skills. We then examined existing workflows using LLMs, indicating areas where they have been or could be applied to materials science research. Next, we demonstrated two example workflows using LLMs, one for 3D microstructure data analysis co-ordination and another for the automated collection of LLM-labelled micrographs from the literature. Finally, we explored various issues and challenges that the current generation of LLMs are yet to resolve, including hallucination and confabulations.

We believe the versatility and emergent properties of LLMs will make them strong tools in an increasingly automated, connected and data-driven research environment. This is doubly true for materials science which must cover a broad range of length-scales, materials, techniques and topics.

At their current stage of development, LLMs are promising tools for accelerating research and exploration, acting as tireless interdisciplinary workers. They must, however, be used with full understanding of their drawbacks – not as infallible generators of new, deep insights, but instead in workflows that minimise and are robust to hallucinations. There is an old saying: “fire is a good servant, but a bad master”.

## Author contributions

GL and RD contributed equally to the work. SJC contributed to the development of concepts presented in all sections of the work and made substantial revisions and edits to the manuscript.

## Conflicts of interest

The authors declare no competing interests.

## Supplementary Material

DD-003-D4DD00074A-s001

## Data Availability

The code needed to run the micrograph scraping, extraction and LLM labelling is available at https://github.com/tldr-group/micrograph_extractor with an MIT license agreement. The scraped dataset of 842 micrographs is available in the above repository in the https://github.com/tldr-group/micrograph_extractor/tree/main/micrographs and the corresponding labels in https://github.com/tldr-group/micrograph_extractor/blob/main/micrographs/labels.csv. The code to run MicroGPT is available at https://github.com/tldr-group/Microgpt.
