# Supplementary material for: Materials science in the era of large language models: a perspective
Source: Digit Discov. 2024 Jun 5;3(7):1257–72. doi: 10.1039/d4dd00074a (PMC13580199; doi:10.1039/d4dd00074a)
Supplement: Supplementary file 1 [file DD-003-D4DD00074A-s001.pdf]

Electronic Supplementary Material (ESI) for Digital Discovery.
This journal is © The Royal Society of Chemistry 2024

Materials science in the era of large language models: a perspective

Preprint - May 31, 2024

Ge Lei † 1, Ronan Docherty † 1, 2, and Samuel J. Cooper * 1

[1] *Dyson School of Design Engineering, Imperial College London, London SW7 2DB*
[2] *Department of Materials, Imperial College London, London SW7 2DB*

Supplementary Information

S1 Extended LLM theory

S1.1 Attention

Attention is implemented with the matrix multiplication [1]

$$\text{Attention}(Q, K, V) = \text{softmax}(\frac{QK^T}{\sqrt{d_k}})V \tag{1}$$

where Q, K, V are all linear projections of the input sequence of tokens X with learnable weight matrices W_Q, W_k and W_v and $\sqrt{d_k}$ is a scaling factor to prevent the dot product getting too large. Vaswani *et. al.*[1] describe it as "a weighted sum of the values, where the weight assigned to each value is computed by a compatibility function of the query with the corresponding key". QK^T describes how important each token in the context is at predicting or representing the value of every other token[2]. This can be interpreted as a differentiable lookup table[3] or learned message passing, with QK^T determining the similarity of the input to the (learned) keys of this table and V being the (learned) values. The values in V are then weighted by this similarity score and passed to the next layer.

The authors also proposed multi-head attention[1], where different heads perform the attention calculation on the same input sequence (again in parallel), which improved the model's ability to focus on multiple positions in a sequence. Without the multiple heads, averaging over different training examples where different positions in the sequence contain the relevant pattern (noun-pronoun, verb-subject, *etc.*) would have diminished this ability.

S1.2 Transformer network architectures

The original transformer network[1] had encoder-decoder architecture, with the encoder formed of N identical encoder layers and the decoder formed of N identical decoder layers. Each encoder layer has multi-headed attention applied to an embedding (vector representation of the tokens in a new subspace) of the input sequence or the previous layer's output, followed by a feed-forward linear layer. The decoder layer has an additional multi-headed attention layer at the start, and 'cross-attention' with the output of nth encoder layer. An example encoder layer is show in Figure 1 .

†These authors contributed equally.
*Corresponding: samuel.cooper@imperial.ac.uk

The original transformer network was an encoder-decoder, which worked well for sequence-to-sequence translation, but not all transformers or LLMs are encoder-decoders. The GPT family [4, 5, 6] are decoder-only, which work better for auto-regressive token generation.

S1.3 LLM output sampling

Various sampling and post-processing strategies exist, which can be combined with temperature and each other. 'Top-k sampling' chooses the best k-possible tokens and only uses them in the softmax, reducing the computational overhead. 'Top-p sampling' chooses from tokens to sample from by summing the n most probable tokens until their cumulative probability is greater than some cutoff p [7].

'Test-time sampling' generates multiple outputs for the same prompt and uses some heuristic like total sequence probability to decide which to return to the user [7]. This is effective but increases the total compute cost. Other ways of improving results (at the cost of added compute) are search strategies, where various next-token options are explored in parallel. A notable example of this strategy is 'beam search'[8].

LLM outputs can be forced to conform to a given format or scheme (like JSON) via 'constraint sampling', which involves filtering output token logits to a list of valid tokens for the scheme and selecting the most probable (*i.e,* a JSON should end in a }) [7]. This form of sampling could prove incredibly useful for writing programs or robotics routines or outputting niche data formats (common in research) that must fit some existing rules. OpenAI have already added a 'JSON mode' to their API for such situations.

S2 Case study 1: automated 3D microstructure analysis

S2.1 Full Prompts

Below are the system prompts for MicroGPT

Prompt 1: MicroGPT system prompt

```
instructions_microgpt = """ You are an assistant to analyze microstructure.
Remember:

1.  You can invoke tools for analysing tomographic data.  For image analysing,
please ensure to call the function once for each path name provided.
Typically, the number of times the function needs to be invoked corresponds directly
to the number of path names you have.
2.  After writing the code, always use a function, create_and_execute_python_file,
to upload and execute it.
3.  If the user ask for anlysis the all images in a specific folder, please
use data_analysis function.  If use ask for analysis an image, please use other
function.
4.  If the user ask to filter data in a dataset, eg.  try to find iron related 3D
images in a specific directory, please use data_filter function.
5.  If the user requests to reuse a tool that is included in a Python file, please
employ the 'tool_reuse' function"""
```

Prompt 2: simulation function

```
delimiter = "####"
```

```
system_message = f""" Follow these steps to answer the customer queries.

Step 1:{delimiter} First, determine if the user is asking a question about analyzing
3D images in a specific directory.  If the user is asking about analyzing 3D images
in a specific directory, call a function to extract the filenames of images in the
directory.
Step 2:{delimiter} Next, call a simulation function to analyze the images.
Step 3:{delimiter} Finally, store all the data in a CSV file.

Use the following format:
Step 1:{delimiter} <step 1 reasoning>
Step 2:{delimiter} <step 2 reasoning>
Step 3:{delimiter} <step 3 reasoning>

Make sure to include {delimiter} to separate every step.  """
```

Prompt 3: data filtering function

```
delimiter = "####"

system_message = f""" Follow these steps to answer the customer queries.

Step 1:{delimiter} First, confirm whether the user is asking you to filter the data
in the database based on their criteria.   Determine the directory of the database.
Step 2:{delimiter} If the user is asking about  filter 3D images in a dataset, you
have a function called find_json, you can use it to unfold zip file, find the meta
data of the dataset in the file and extract the metadata
Step 3:{delimiter} Now that you have the metadata, which is in a JSON format, focus
on the description and keywords within the metadata.  Filter all the data in the
database that aligns with the user's criteria.

Use the following format:
Step 1:{delimiter} <step 1 reasoning>
Step 2:{delimiter} <step 2 reasoning>
Step 3:{delimiter} <step 3 reasoning>

Make sure to include {delimiter} to separate every step.  """
```

Prompt 4: tool reuse function

```
delimiter = "####"

system_message = f""" Follow these steps to answer the customer queries.

Step 1:{delimiter} First, confirm whether the user wants to reuse the tool created
before, by modifing the code of a file according to his needs and then run it.  If
yes, call the read_file function to read this file, specifying the file path clearly
Step 2:{delimiter} Now that you have the code, make modifications according to the
user's requirements.
Step 3:{delimiter} Call the create_and_execute_python_file function to save and
```

```
overwrite the original code, keeping the file name unchanged,clearly specifying the
file path to be saved.

Use the following format:
Step 1:{delimiter} <step 1 reasoning>
Step 2:{delimiter} <step 2 reasoning>
Step 3:{delimiter} <step 3 reasoning>

Make sure to include {delimiter} to separate every step.  """
```

S2.2 An example interaction

The following is a conversation between the user and MicroGPT regarding the Microlib Dataset. These dialogues the following capabilities of MicroGPT: data collection, custom tool creation and reuse, data filtering, data simulation, data analysis, data visualization, etc.

Data Collection

```
User:  Can you search for the Microlib online, which is a dataset of 3D
microstructures?

Assistant:  The Microlib dataset of 3D microstructures is available on Zenodo.  Here
are some of the relevant records:
1.  [Zenodo Record 7118559](https://zenodo.org/records/7118559)
2.  [Zenodo Record 4047774](https://zenodo.org/records/4047774)
3.  [Zenodo Record 7867413](https://zenodo.org/records/7867413)
4.  [Zenodo Record 8112442](https://zenodo.org/records/8112442)
5.  [Zenodo Record 7660542](https://zenodo.org/records/7660542)
6.  [Zenodo Record 7043464](https://zenodo.org/records/7043464)
7.  [Zenodo Record 5011434](https://zenodo.org/records/5011434)
8.  [Zenodo Record 5090057](https://zenodo.org/records/5090057)
9.  [Zenodo Record 7702179](https://zenodo.org/records/7702179)
10.  [Zenodo Record 7820168](https://zenodo.org/records/7820168)
You can visit these links to find more information about each dataset and access the
data.

User:  Download the first one zenodo record 7118559'

Assistant:  Do you want to download the file from
https://zenodo.org/records/7118559/files/
microlibDataset.zip?download=1?  (yes/no):  yes
File downloaded:  microlibDataset.zip
The dataset from Zenodo record 7118559 has been downloaded successfully as
'microlibDataset.zip'.  If you would like to proceed with extracting and organizing
the files, or any other action with this dataset, please let me know!
```

Custom Tool Creation and Reuse

```
User: Please write and execute a script to unzip the file './microlibDataset.zip

Assistant: Python script saved as created_script.py Do you want to execute the
script? (yes/no):yes
I have written and executed a Python script to unzip the file 'microlibDataset.zip'.
The contents have been successfully extracted. If you need to work with these files
or have any other requests, please let me know!

User: Please unzip the file named './Test_tool_reuse.zip' using the tool in
'./created_script.py

Assistant:
============================Thinking============================
Step 1:#### To unzip the file './Test_tool_reuse.zip', we first need to read the
existing Python script located at './created_script.py'. We will use the read_file
function to read the content of this file. This will allow us to understand the
current functionality of the script and modify it to include the code necessary for
unzipping the file.
Step 2:#### After reading the file, we need to modify the code to add the
functionality to unzip files. We will import the 'zipfile' module and use the
'ZipFile' class along with the 'extractall' method to unzip the file. The code
modification will include handling the path of the zip file and the destination
directory where the contents will be extracted.
Step 3:#### Once the code has been modified to include the unzip functionality,
we will call the create_and_execute_python_file function to save and overwrite
the original code in './created_script.py'. This will update the script with the
new functionality, and then we will execute the script to perform the unzipping
operation.
========================Start solving the problem!=================
Python script saved as created_script.py Do you want to execute the script?
(yes/no): yes
The files have been extracted to: ./Test_tool_reuse
The script has been updated to unzip the file 'Test_tool_reuse.zip', and the
execution of the script was successful. The files have been extracted to a
directory named 'Test_tool_reuse'
```

Data Filter

```
User: In the 'microlibDataset.zip' file, can you find all the 3D images related to
cast iron?

Assistant:
============================Thinking============================
Step 1:#### The user is asking to filter the data in the database based on their
criteria, which is to find all the 3D images related to cast iron. The directory of
the database is indicated as 'microlibDataset.zip'.
Step 2:#### Since the user is asking to filter 3D images in a dataset, I will use
the function find_json to unfold the 'microlibDataset.zip' file, find the metadata
of the dataset in the file, and extract the metadata.
Step 3:#### Now that I have the content of metadata in json-format, I will focus
on the description and keywords in the metadata, and filter all the data in the
database which align with the user's criteria, which in this case is "cast iron".
========================Start solving the problem!=================
```

```
Found file:  data_anns.json.  Is this the metadata file?  (yes/no):yes
JSON file contains items with the following fields:
name
link_doitpoms
data_2D
data_2D_original
data_3D
preview
inpaint_movie
long_movie
data_type
brief_description
keyword
category
element
technique
long_description
contributor
organisation
Enter the fields you want to use to filter your data (separated by
commas):name,keyword
Based on the metadata extracted from the 'microlibDataset.zip' file, we can filter
all the data related to "cast iron." Here are the entries that match the criteria:
- Microstructure 066:  Keywords including "cast iron" - Microstructure 068:
Keywords including "cast iron" - Microstructure 072:  Keywords including "cast
iron" - Microstructure 360:  Keywords including "cast iron" - Microstructure 365:
Keywords including "white cast iron" - Microstructure 368:  Keywords including
"white cast iron" - Microstructure 370:  Keywords including "white cast iron" -
Microstructure 372:  Keywords including "white cast iron" - Microstructure 376:
Keywords including "white cast iron" - Microstructure 378:  Keywords including
"grey cast iron" - Microstructure 381:  Keywords including "grey cast iron" -
Microstructure 387:  Keywords including "grey cast iron" - Microstructure 393:
Keywords including "cast iron" - Microstructure 396:  Keywords including "cast
iron" - Microstructure 406:  Keywords including "cast iron" - Microstructure 782:
Keywords including "cast iron" - Microstructure 784:  Keywords including "cast iron"
- Microstructure 797:  Keywords including "cast iron"
These entries are filtered out because they contain the keyword "cast iron" or a
variation thereof, such as "white cast iron" or "grey cast iron." If you need more
information about these microstructures or if you need to extract specific files
related to these entries, please let me know.
Please input an exmaple of data filename:  microstructure001.tif
Based on the example filename 'microstructure001.tif' provided by the user,
we can deduce that the data filenames in the dataset follow the pattern
'microstructureXYZ.tif', where 'XYZ' represents a three-digit number, covering all
the ones in the dataset.
Files with the pattern mentioned above have been extracted successfully.  However,
since I do not have direct access to the file system to list all the filenames, I
will now list the filenames based on the metadata provided earlier that are related
to "cast iron":
1.  microstructure066.tif 2.  microstructure068.tif 3.  microstructure072.tif
4.  microstructure360.tif 5.  microstructure365.tif 6.  microstructure368.tif
7.  microstructure370.tif 8.  microstructure372.tif 9.  microstructure376.tif 10.
microstructure378.tif 11.  microstructure381.tif 12.  microstructure387.tif 13.
microstructure393.tif 14.  microstructure396.tif 15.  microstructure406.tif 16.
microstructure782.tif 17.  microstructure784.tif 18.  microstructure797.tif
```

```
These files are stored in a data folder and are ready for any further analysis or
review that you may need.  If you'd like to proceed with any specific analysis or
have other requests, please let me know!
```

Data Simulation

```
User:  Could you analyze the 3D images in the './data' folder to determine their
tortuosity, diffusion factor, volume fraction, and surface area?

Assistant:
=========================Thinking...=======================
Step 1:  #### The user is asking to analyse 3D images in a specific directory
('./data').  Therefore, I will call a function to extract the filenames of images
in that directory.
Step 2:  #### Now that we have the filenames, I will call a simulation function to
analyse the images for tortuosity, diffusion factor, volume fraction, and surface
area.
Step 3:  #### After the analysis is complete, I will store all the data into a CSV
file.
=================Start solving the problem!================
The image paths from the specified directory ('./data') are as follows:
- './data/microstructure368.tif' - './data/microstructure378.tif' -
'./data/microstructure782.tif' - './data/microstructure370.tif' -
'./data/microstructure365.tif' - './data/microstructure387.tif' -
'./data/microstructure797.tif' - './data/microstructure360.tif' -
'./data/microstructure376.tif' - './data/microstructure396.tif' -
'./data/microstructure372.tif' - './data/microstructure406.tif' -
'./data/microstructure066.tif' - './data/microstructure072.tif' -
'./data/microstructure784.tif' - './data/microstructure393.tif' -
'./data/microstructure068.tif' - './data/microstructure381.tif'
The simulation results for the analysis of the 3D images are as follows:
1.  Microstructure368.tif:  - Effective Diffusivity:  0.4152 - Tau (Tortuosity
Factor):  1.8422 - Volume Fraction:  [0.2351, 0.7649] - Surface Area:  0.0919
2.  Microstructure378.tif:  - Effective Diffusivity:  0.3918 - Tau:  1.9773 - Volume
Fraction:  [0.2252, 0.7748] - Surface Area:  0.1074
3.  Microstructure782.tif:  - Effective Diffusivity:  0.3223 - Tau:  1.7827 - Volume
Fraction:  [0.4255, 0.5745] - Surface Area:  0.0396
4.  Microstructure370.tif:  - Effective Diffusivity:  0.0464 - Tau:  4.2417 - Volume
Fraction:  [0.8031, 0.1969] - Surface Area:  0.0838
...  (summary continues for each image)
One of the images, Microstructure797.tif, encountered an issue and could not be
processed.
The data from the analysis has been successfully stored into a CSV file.  You can
download it using the following link:
[Download microstructure_analysis_results.csv]
(sandbox:/mnt/data/microstructure_analysis_results.csv)
```

Table S1: Cast iron related microstructure's simulation results in Microlib

Microstructure	Effective Diffusivity	Tortuosity	Surface Area	Volume Fraction Phase 1	Volume Fraction Phase 2
microstructure368.tif	0.4152	1.8422	0.0919	0.2351	0.7649
microstructure378.tif	0.3918	1.9773	0.1074	0.2252	0.7748
microstructure782.tif	0.3223	1.7827	0.0396	0.4255	0.5745
microstructure370.tif	0.0464	4.2417	0.0838	0.8031	0.1969
microstructure365.tif	0.1709	2.0907	0.0429	0.6427	0.3573
microstructure387.tif	0.208	2.8765	0.1266	0.4016	0.5984
microstructure360.tif	0.024	5.9349	0.0657	0.8575	0.1425
microstructure376.tif	0.1422	2.5106	0.1341	0.6431	0.3569
microstructure396.tif	0.6924	1.1616	0.0425	0.1958	0.8042
microstructure372.tif	0.0012	85.388	0.0855	0.9013	0.0987
microstructure406.tif	0.3071	1.7043	0.0908	0.4765	0.5235
microstructure066.tif	0.1644	2.1616	0.0919	0.6447	0.3553
microstructure072.tif	0.2527	2.4606	0.1444	0.3783	0.6217
microstructure784.tif	0.0751	5.1606	0.1768	0.6122	0.3878
microstructure393.tif	0.148	2.7249	0.0614	0.5966	0.4034
microstructure068.tif	0.2682	2.17	0.0898	0.418	0.582
microstructure381.tif	0.4692	1.7356	0.1085	0.1858	0.8142

Data Analysis

```
User:  Review and compare these data to identify any data points that are
conspicuously unreasonable or significantly divergent from the rest.

Assistant:  Based on the analysis of the dataset for outliers, we've identified the
following:
1.  **microstructure396.tif**:  The effective diffusivity of 0.6924 is identified
as an outlier, as it is significantly higher than what we would expect compared to
other data points.
2.  **microstructure372.tif**:  This microstructure has an extraordinarily high
tortuosity (Tau) value of 85.388, which is significantly higher than any other data
point, indicating that this value is an outlier and warrants further investigation.
3.  **microstructure784.tif**:  The surface area value of 0.1768 is marked an
outlier on the higher end, meaning it is significantly larger than most other
surface area values, possibly indicating a highly complex interface between phases.
The rest of the data points appear reasonable, with volume fractions summing close
to 1 as they should.  It's important to remember that while the statistical method
used here highlights significant deviations from the mean, it cannot conclusively
determine whether a value is truly unreasonable without additional context regarding
the materials and processes involved.  These identified outliers should be reviewed
carefully with consideration of the experimental conditions or possibly re-measured
if experimental error is suspected.

User:  I believe that only the data for microstructure372 is incorrect.  Given
that there is an issue with this data, please disregard microstructure 372 when
addressing the following question.  Analyze which microstructure possesses the
highest tortuosity.
```

```
Assistant:  After correctly excluding the data for microstructure372, the
microstructure with the highest tortuosity is microstructure360, which has a
tortuosity (Tau) value of 5.9349.

User:  Please compare microstructure 393, 368, and 365

Assistant:  Here is a comparison of the specified microstructures based on their
properties:
### Effective Diffusivity (D_eff):
- **microstructure393.tif**:  Has a moderate effective diffusivity of around 0.148.
- **microstructure368.tif**:  Exhibits the highest effective diffusivity among the
three, at about 0.415.
- **microstructure365.tif**:  Close to microstructure393.tif, with a value of around
0.171.
### Tortuosity (Tau):
- **microstructure393.tif**:  Has the highest tortuosity with a value of
approximately 2.725.
- **microstructure368.tif**:  Has the lowest tortuosity, with a value close to
1.842.
- **microstructure365.tif**:  Has a tortuosity value around 2.091.
### Surface Area:
- **microstructure393.tif**:  Has a moderate surface area valued at approximately
0.061.
- **microstructure368.tif**:  Has the highest surface area with a value of around
0.092.
- **microstructure365.tif**:  Has the lowest surface area, near 0.043.
### Volume Fraction Phase 1:
- **microstructure393.tif**:  Has a reasonably high volume fraction phase 1 of
approximately 0.597.
- **microstructure368.tif**:  Has the lowest volume fraction phase 1 of about 0.235.
- **microstructure365.tif**:  Has the highest volume fraction phase 1, around 0.643.
### Volume Fraction Phase 2 (complementary to Phase 1):
- **microstructure393.tif**:  Has the lowest volume fraction phase 2 of about 0.403.
- **microstructure368.tif**:  Has the highest volume fraction phase 2 of
approximately 0.765.
- **microstructure365.tif**:  Has a moderate volume fraction phase 2, around 0.357.
Overall, **microstructure368.tif** stands out due to its high effective diffusivity
and surface area, which are beneficial for a catalyst carrier that facilitates
reaction and filtration.  However, **microstructure365.tif** also has desirable
properties, such as the highest volume fraction phase 1, which might imply a more
solid structure possibly beneficial for mechanical stability in a catalyst support.
**Microstructure393.tif** appears to be a middle ground with moderate properties
across the board.
```

Data Visualization

```
User:  Disregard the unreasonable data, can you generate some figures to create
visualizations for the data?  Histograms for each numerical column to understand
the distribution of values.  Scatter plots to explore relationships between pairs of
numerical variables (e.g., Effective Diffusivity vs.  Tortuosity)

Assistant:  Here are the visualizations for the data:
```

```
### Histograms for Each Numerical Column - The histograms show the distribution
of values for each numerical variable:  Effective Diffusivity, Tortuosity, Surface
Area, Volume Fraction Phase 1, and Volume Fraction Phase 2.
### Scatter Plots for Relationships Between Pairs of Numerical Variables - The
scatter plots explore relationships between pairs of numerical variables, such as:
1.  Effective Diffusivity vs.  Tortuosity
2.  Effective Diffusivity vs.  Surface Area
3.  Effective Diffusivity vs.  Volume Fraction Phase 1
4.  Tortuosity vs.  Surface Area
5.  Tortuosity vs.  Volume Fraction Phase 1
6.  Surface Area vs.  Volume Fraction Phase 1
These figures provide insights into how the various microstructural properties are
distributed and how they may relate to one another within the dataset.
```

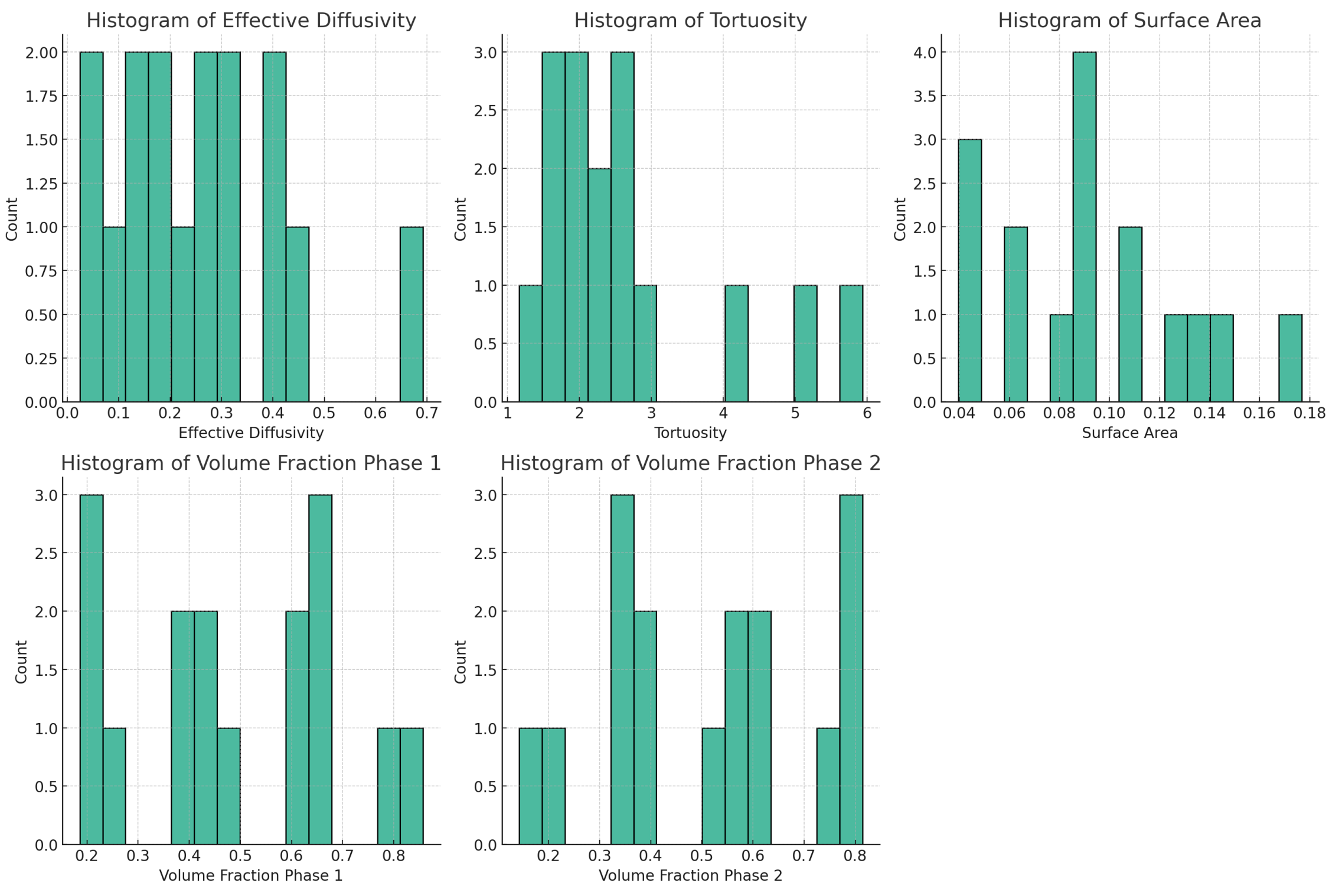

Figure S1: Histograms of effective diffusivity, tortuosity, surface area, and volume fraction of cast-iron microstructures in Microlib as returned by MicroGPT.

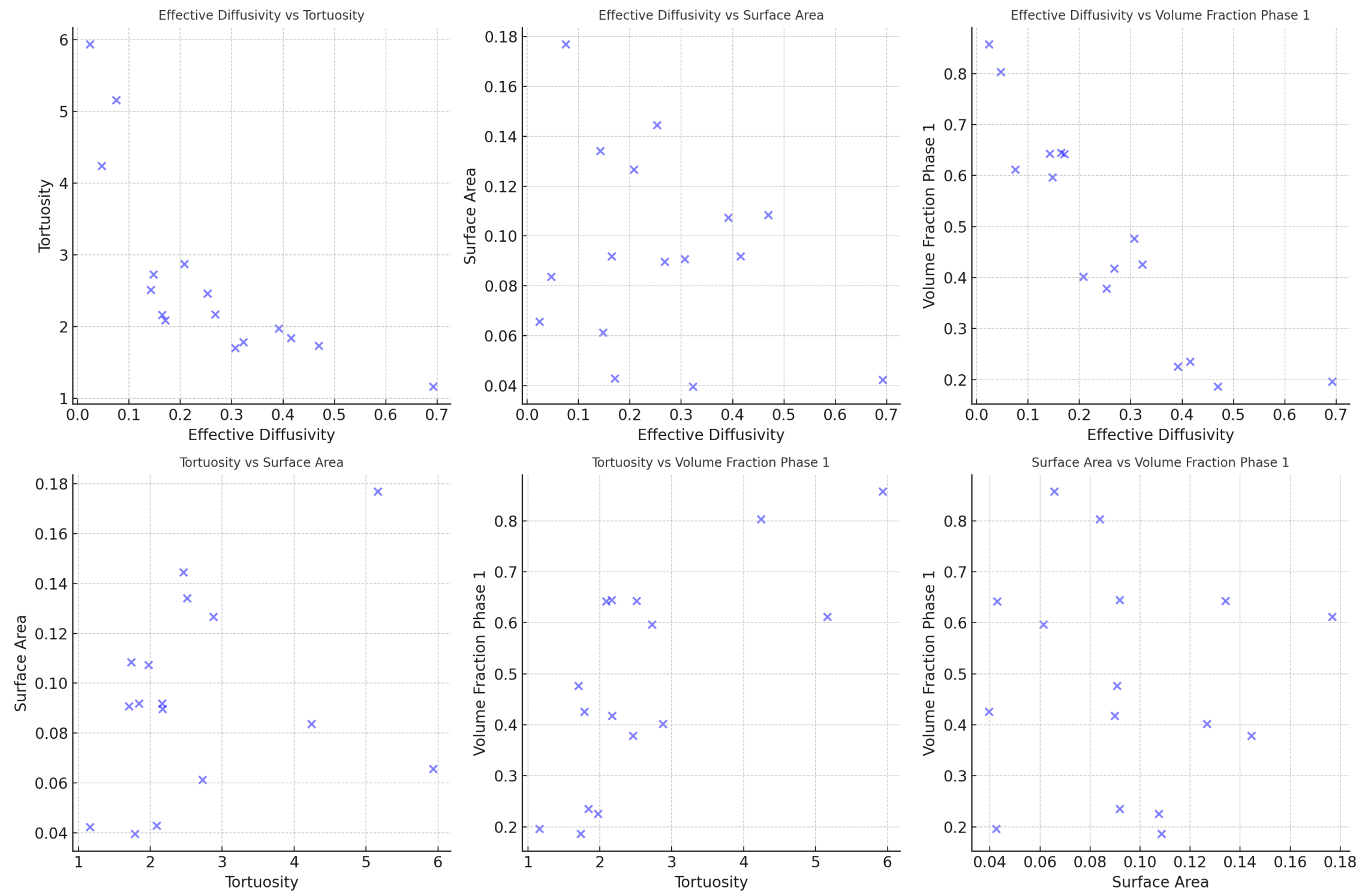

Figure S2: Scatter plots exploring the relationships between pairs of numerical variables of cast-iron microstructures in Microlib as returned by MicroGPT.

S3 Case study 2: automated labelled microstructure dataset collection

S3.1 Scraping and figure extraction

The metadata (title, abstract, authors, DOI, *etc.*) of 14,000 papers matching the search query "`microscopy`" were scraped from arXiv and chemrXiv via their APIs. A subset of 500 of these were downloaded from chemrXiv and the figures and their matching captions extracted with 'pdffigures2.0'[9]. This worked well but the tool sometimes failed to extract the whole figure or caption.

Figures were split into subfigures by detecting whitespace pixels (pixels whose intensity ≈ 255) and thresholding. The binary image was dilated to remove small gaps. The connected components of the non-whitspace pixels were computed and bounding boxes generated - these were then saved as subfigures. Small figures like sub-figure letter captions, were discarded. This process works under the assumption that figures use around 5px whitespace to separate subfigures, which tended to be true in most cases.

S3.2 LLM labelling

We chose to use GPT3.5 and GPT4 as the LLM and VLM in the study, primarily for convenience and performance. Given the relatively uncomplicated nature of the task, a local LLM would be preferable for running this workflow at-scale. The prompts used for each step are in Section S3.5.

The caption and abstract text-only GPT4 worked well for detecting and categorization of figures (see Section S3.4), but in order to detect if a specific subfigure was a micrograph a vision model was needed.

S3.3 Evaluation

A custom labelling GUI was written to label figures and subfigures in papers in terms of whether they had a micrograph present, what material was shown and what instrument was used to capture the micrograph. It also

allowed comparison of the human labels to the LLM labels for evaluation. We chose to do labelling before evaluation (*i.e,* a two-step process) rather than just reviewing the LLM answers in order to avoid bias.

For the VLM evaluation, we noted the lack of false positives by examining the figures in the produced dataset and resulting T-SNE plot, and not via the custom GUI. This method did not allow us to report the full confusion matrix.

S3.4 Performance and issues

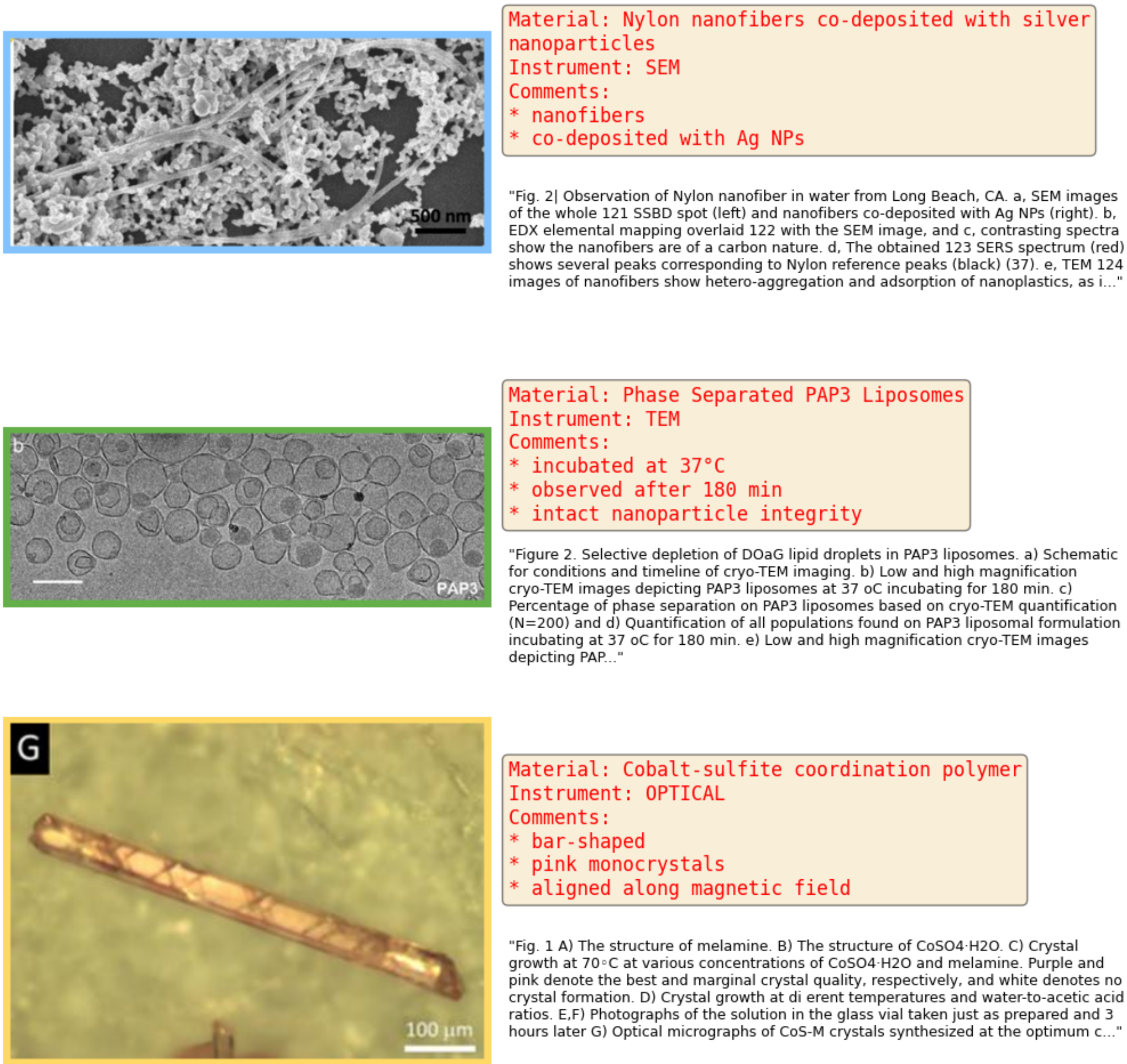

Figure S3: Examples of GPT-4V labels for micrographs and captions randomly sampled from the dataset. The border colour coding is the same as Figure 6. There is a good agreement between the captions and the LLM labels & comments, though we note there can be overlap between the comments and the label, and that sometimes important information can be missed *i.e,* the 'Cobalt-suflite coordination polymer' is potentially better described as a 'Cobalt-suflite coordination polymer crystal'. This is indicative of the lack of true domain knowledge or understanding - the LLM is able to competently parse the caption (and abstract), extract the material of interest from context but fails to give a complete, physically-grounded description. As a VLM, GPT4-V was sometimes able to match the sub-figure caption (like 'b.' or 'a)') in the image to the correct part of the caption when generating labels and comments, which is a useful capability when multiple different materials are included in a single figure.

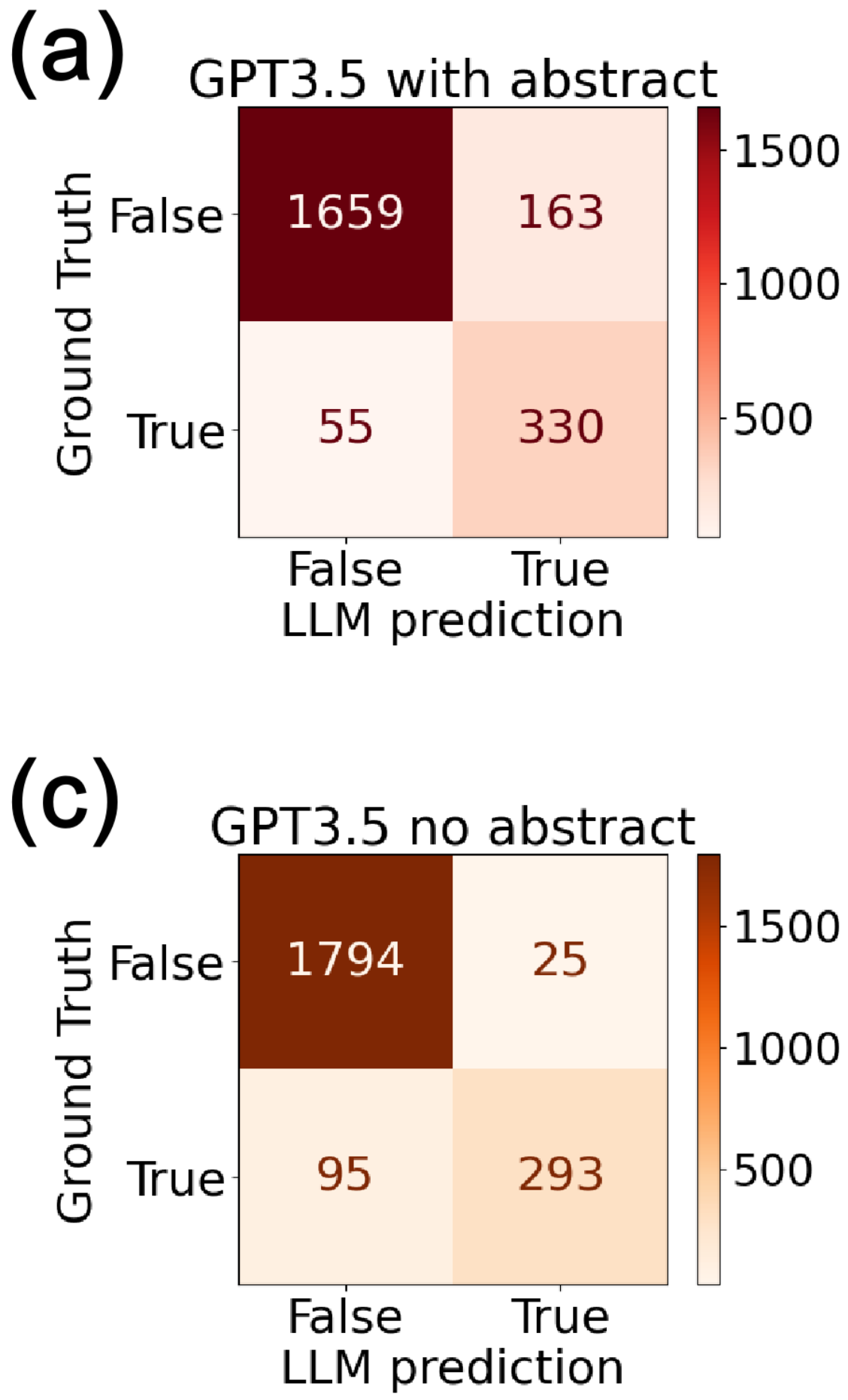

Figure S4: Confusion matrices of micrograph detection for **(a)** GPT3.5 with caption & abstract, **(b)** GPT4 with caption & abstract, **(c)** GPT3.5 with caption & no abstract and **(d)** GPT4 with caption & no abstract. GPT4 with abstract performs the best, with the highest sensitivity and specificity.

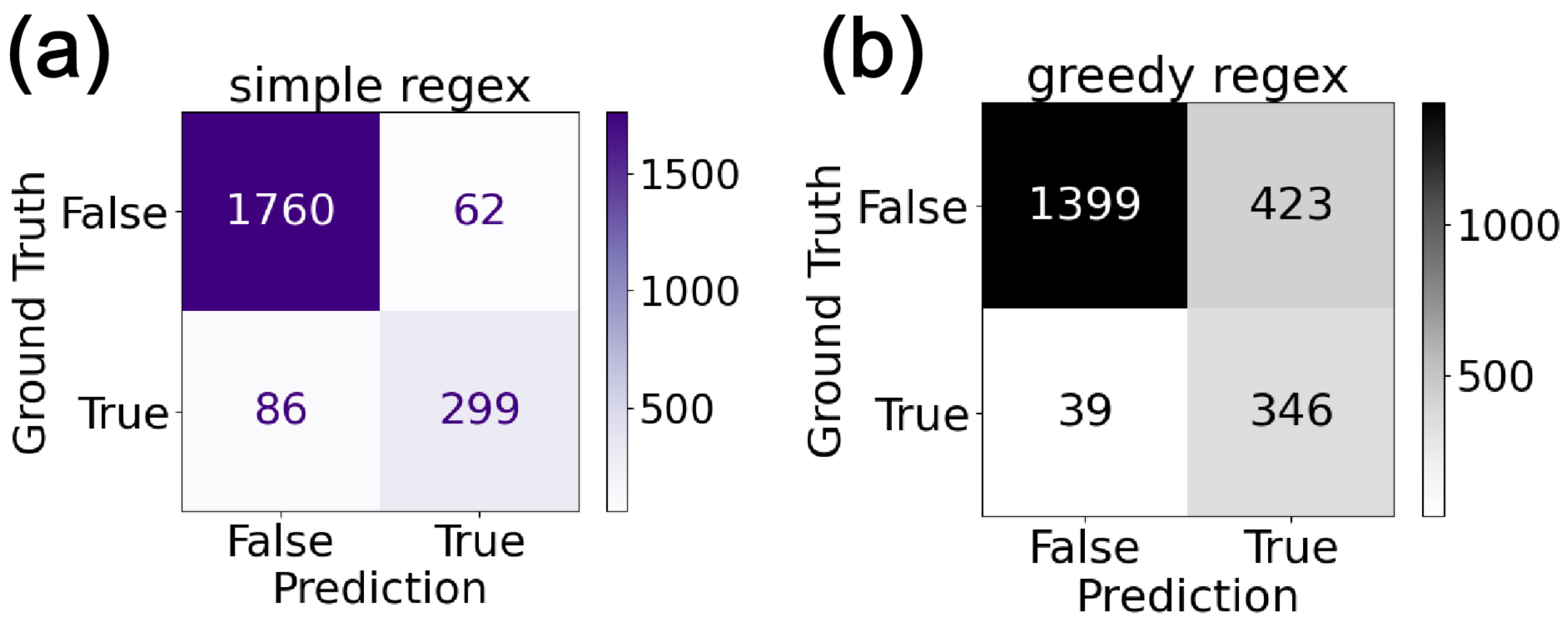

Figure S5: Confusion matrices of micrograph detection for two string matching (regex) approaches. **(a)** is the simple regex scheme, where a figure is labelled as containing a micrograph if it contains the phrase 'image' or 'micrograph'. **(b)** is the greedy scheme, where a figure is labelled as containing a micrograph if it matches the simple scheme or contains the name of a microscopy technique like 'SEM' or 'AFM'. The simple approach works well though has less true positives than LLM-based results where the caption is more ambiguous. As expected, the greedy approach has more true positives but far more false positives.

S3.5 Prompts

Prompt 1: LLM micrograph screening utilizing only the captions of figures as input

```
system_message ="""

You are an expert on micrographs, images captured using microscopes such as SEM,
TEM, or AFM, etc.

Given caption of a figure from an academic paper, answer the question:
Is there a micrograph in this figure?  Respond with 'true' or 'false'

IMPORTANT: The anwer should only contain pure JSON format only with the fields
"isMicrograph"

Here's an example of output:

{
"isMicrograph":  "true or false"
}

"""
```

Prompt 2: LLM micrograph screening utilizing the captions of figures and paper abstract as input

```
system_message ="""
```

```
You are an expert materials scientist.  You study micrographs, which are images
taken using a microscope.

Based on an academic paper's abstract and a specific figure's caption, provide
answers in JSON format to the following questions:

1.  Is there a micrograph in this specific figure?  Answer with 'true' or 'false'.
2.  If a micrograph is present, list the techniques used in this figure (e.g., SEM,
TEM, Optical Microscopy).  Note that techniques mentioned in the abstract might not
be used in this figure.
3.  If a micrograph is present, list the the full name of materials depicted in the
micrographs e.g., 'Lithium Nickel-Manganese-Cobalt (NMC) 811 cathode' or 'Insulin
aggregates'.
4.  Are there any noteworthy details about the micrograph, such as unique
processing conditions or observed anomalies, in a series of brief phrases (e.g.,
['heat-treated', 'cracked', 'sintered'])?

Here's an example of how the JSON output should look with micrograph present:

{
"isMicrograph":  "true",
"instrument":  "Technique",
"material":  "Description",
"comments":  ["comment1", "comment2", "comment3"]
}

Here's an example of how the JSON output should look without micrograph present:

{
"isMicrograph":  "false"
}

IMPORTANT: The answer should only contain pure JSON data matching the fields
provided in the examples.
"""
user_message = f""" The abstract is:  {abstract_escaped}, and the captions are:
{captions_escaped}"""
```

Prompt 3: VLM micrograph recognition with a single sub-figure as input

```
user_message_1 = """

You are an expert materials scientist specializing in micrographs.
Typically, these are greyscale and might include annotations like scale bars or
arrows, although not always.

Your task:Given a subfigure image from a research paper
1.  check if it is a micrograph or another type of figure, such as a graph or
diagram
2.  check if it includes sub-subfigures.

If the image is solely a micrograph without any sub-subfigures, you should respond
with 'TRUE';
```

```
otherwise, respond with 'FALSE'.

Here is an example of output:
"Reason: The image is a micrograph with 2 sub-subfigures.
Answer: FALSE"

"""
```

Prompt 4: VLM micrograph recognition with a main figure and a sub-figure as input

```
system_message = """

You are an expert materials scientist working on micrographs. The first image is a
main image is taken from a research paper. The second image is a subfigure cropped
from the main image. It is might be a micrograph.

Focus on the abstract of the paper, captions, and the content of these images.
Answer the questions below:
1.Is the cropped image a micrograph? Answer with a single 'true' or 'false'.
2.What technique (e.g., SEM, TEM) was used to create the micrograph in the cropped
image? Provide a brief answer, such as 'SEM' or 'Optical Microscopy'.
3.What material is shown in the micrograph? Provide the full name e.g., 'Lithium
Nickel-Manganese-Cobalt (NMC) 811 cathode' or 'Insulin aggregates'.
4.If there are any interesting things about the micrograph, like specific processing
conditions or anomalies, put these in a list of single phrases (e.g ['heat-treated,
'cracked', 'sintered']).
5.Which part of the caption of the mainfigure does this subfigure correspond to?
Extract the caption of the subfigure, and its label e.g (a), (b) if possible.

Here's an example of the JSON output format:

{
"isMicrograph": True,
"instrument": "Technique",
"material": "Description",
"comments": ["comment1", "comment2", "comment3"]
"subfigure_caption": "(label): caption of subfigure"
}

IMPORTANT: The answer should only contain pure JSON data matching the fields
provided in the examples.

"""

user_message = f"The abstract is: {abstract_escaped}, and the captions are:
{captions_escaped}"
```

References

[1] Ashish Vaswani et al. "Attention is all you need". In: *Advances in neural information processing systems* 30 (2017).
[2] Mary Phuong and Marcus Hutter. "Formal Algorithms for Transformers". In: *arXiv preprint arXiv:2207.09238* (2022).
[3] Brandon Rohrer. https://e2eml.school/transformers.html.
[4] Alec Radford et al. "Improving language understanding by generative pre-training". In: (2018).
[5] Tom B. Brown et al. "Language Models are Few-Shot Learners". In: *arXiv preprint arXiv:2005.14165* (2020).
[6] OpenAI. "GPT-4 Technical Report". In: *arXiv preprint arXiv:2303.08774* (2023). arXiv: 2303.08774 [cs.CL].
[7] Chip Huyen. *Sampling for Text Generation*. URL: https://huyenchip.com/2024/01/16/sampling.html.
[8] Markus Freitag and Yaser Al-Onaizan. "Beam Search Strategies for Neural Machine Translation". In: *Proceedings of the First Workshop on Neural Machine Translation*. Association for Computational Linguistics, 2017. DOI: 10.18653/v1/w17-3207. URL: http://dx.doi.org/10.18653/v1/W17-3207.
[9] Christopher Clark and Santosh Divvala. "PDFFigures 2.0: Mining figures from research papers". In: *2016 IEEE/ACM Joint Conference on Digital Libraries (JCDL)*. 2016, pp. 143–152.
